# Characterization of *Komagataeibacter* Isolate Reveals New Prospects in Waste Stream Valorization for Bacterial Cellulose Production

**DOI:** 10.3390/microorganisms9112230

**Published:** 2021-10-26

**Authors:** Pietro Cannazza, Antti J. Rissanen, Dieval Guizelini, Pauli Losoi, Essi Sarlin, Diego Romano, Ville Santala, Rahul Mangayil

**Affiliations:** 1 Department of Food, Environmental and Nutritional Sciences (DeFENS), University of Milan, Via Celoria 2, 20133 Milan, Italy; diego.romano@unimi.it; 2 Faculty of Engineering and Natural Sciences, Tampere University, 33720 Tampere, Finland; antti.rissanen@tuni.fi (A.J.R.); pauli.losoi@tuni.fi (P.L.); essi.sarlin@tuni.fi (E.S.); ville.santala@tuni.fi (V.S.); 3 Graduate Program in Bioinformatics, Sector of Professional and Technological Education, Federal University of Parana (UFPR), Curitiba 81520-260, PR, Brazil; dievalg@gmail.com

**Keywords:** *Komagataeibacter rhaeticus*, bacterial cellulose, crude glycerol, minimal medium, whole-genome analysis, acetate

## Abstract

*Komagataeibacter* spp. has been used for the bioconversion of industrial wastes and lignocellulosic hydrolysates to bacterial cellulose (BC). Recently, studies have demonstrated the capacity of *Komagataeibacter* spp. in the biotransformation of inhibitors found in lignocellulosic hydrolysates, aromatic lignin-derived monomers (LDMs) and acetate. In general, detoxification and BC synthesis from lignocellulosic inhibitors requires a carbon flow from acetyl-coA towards tricarboxylic acid and gluconeogenesis, respectively. However, the related molecular aspects have not yet been identified in *Komagataeibacter* spp. In this study, we isolated a cellulose-producing bacterium capable of synthesizing BC in a minimal medium containing crude glycerol, a by-product from the biodiesel production process. The isolate, affiliated to *Komagataeibacter* genus, synthesized cellulose in a minimal medium containing glucose (3.3 ± 0.3 g/L), pure glycerol (2.2 ± 0.1 g/L) and crude glycerol (2.1 ± 0.1 g/L). Genome assembly and annotation identified four copies of bacterial cellulose synthase operon and genes for redirecting the carbon from the central metabolic pathway to gluconeogenesis. According to the genome annotations, a BC production route from acetyl-CoA, a central metabolic intermediate, was hypothesized and was validated using acetate. We identified that when *K. rhaeticus* ENS9b was grown in a minimal medium supplemented with acetate, BC production was not observed. However, in the presence of readily utilizable substrates, such as spent yeast hydrolysate, acetate supplementation improved BC synthesis.

## 1. Introduction

Bacterial cellulose (BC), the nanofibrillar form of cellulose, is synthesized by bacteria of diverse genera, among which the most efficient producers are found in the genus *Komagataeibacter* (formerly *Acetobacter* and *Gluconacetobacter*). In general, BC biogenesis is commenced with the substrate synthesis, UDP-glucose, from the glycolytic pathway (via the catalytic activities of glucokinase, phosphoglucomutase and UDP-glucose pyrophosphorylase). BcsA, BcsB, BcsD, and BcsC proteins (encoded by the bacterial cellulose synthase (bcs) operon) catalyze the polymerization of UDP-glucose to β-1,4-glucan units, transport of the synthesized glucan chain through the periplasmic space, formation of crystalline regions and extracellular export of the synthesized polysaccharide, respectively [1].

BC is a versatile biopolymer with unique characteristics such as biodegradability, purity (not complexed with lignin, hemicellulose or pectin) and superior material properties. Thus, BC is extensively studied for its use in various applications [2,3,4]. Despite the versatile characteristics, studies to improve the production metrics and alternative carbon sources for BC production have been investigated [5,6]. To surpass the low production metrics, rational strategies to engineer the cellulose production machinery and optimize carbon flow through the cellulose synthesis pathway have been conducted in *Komagataeibacter* spp. [3,7,8]. By employing residual carbon sources from agriculture and industries, researchers have coupled BC production with strategic waste disposal [9,10,11,12,13,14]. For instance, Wu et al. (2019) reported a BC titer of ~1.5 g/L from *A. xylinum* ATCC 23767 using crude glycerol, a by-product of biodiesel production from kitchen waste [9]. In a study from Leif Jönsson’s group, BC production by *K. xylinus* ATCC 23770 was investigated in a medium containing three-fold diluted detoxified spruce hydrolysate [11]. The researchers identified that the BC production was absent in cultivation medium with non-detoxified hydrolysate. Detoxification with activated charcoal removed ~94% of furan aldehydes (hydroxymethyl furfural and furfural) and ~60–70% of aliphatic acids (acetic acid and formic acid) from the spruce hydrolysate, enabling *K. xylinus* ATCC 23770 to produce 8.2 g/L BC [11]. However, in these aforementioned studies the waste compounds were supplemented to a rich growth medium containing carbon sources, restricting an exact elucidation of the substrate’s contribution towards BC synthesis [15]. Chemically defined media have been used to circumvent the requirement and influence of other carbon-containing compounds in growth and BC production by *Komagataeibacter* spp. [16,17,18]. In our recent study, a BC titer of 2.9 ± 0.3 g/L was obtained from a *Komagataeibacter* isolate statically grown in MA/9 minimal medium containing crude glycerol [19].

In the present study, we report isolation, biochemical characterization and phylogenetic analysis of a *Komagataeibacter* strain isolated from Kombucha SCOBY (symbiotic colony of bacteria and yeast). The study progresses by comparing the strain’s BC production metrics from glucose, pure glycerol and crude glycerol supplemented to both complex and minimal media. The genome of the strain was sequenced and assembled, and the genetic insights related to carbohydrate uptake mechanisms, BC biogenesis and gluconeogenesis are reported. Taking the genome insights, carbon redirection from pyruvate metabolism towards BC production, via acetate supplementation to complex medium and minimal medium, together with baker’s yeast hydrolysate was investigated.

## 2. Materials and Methods

### 2.1. Materials and Chemicals

Kombucha SCOBY (symbiotic colony of bacteria and yeast) was obtained from Sri Dhanvanthiri Probiotics Ltd., Kodaikanal, India (True Brew Kombucha tea). Sodium chloride, sodium molybdate, potassium chloride, di sodium hydrogen phosphate, dipotassium hydrogen phosphate, potassium dihydrogen phosphate, calcium carbonate, calcium chloride, magnesium sulfate, disodium hydrogen phosphate, citric acid, bromothymol blue and oxidase test disks (product no: 70439) were purchased from Merck (Darmstadt, Germany). Acetic acid and agar were purchased from Fisher Scientific (Loughborough, UK). Glucose and casein amino acids were purchased from VWR International (Leuven, Belgium). Tryptone, peptone and yeast extract were from Lab M Limited (Heywood, UK). Ethanol was from Altia Oyj (Helsinki, Finland). Cycloheximide (Product no: C7698), and cellulase from *Trichoderma reesei* ATCC 26921 (product no: C2730) was purchased from Sigma-Aldrich (St. Louis, MO, USA). GeneJET Genomic DNA Purification Kit was purchased from Thermo Scientific (Waltham, MA, USA). Crude glycerol was generously provided by Perstorp AB (Malmo, Sweden).

### 2.2. Isolation, Culturing and Characterization of Bacterial Cellulose (BC)-Producing Isolates

Cellulose-producing bacteria were isolated from Kombucha SCOBY of Indian origin (True Brew Kombucha, Sri Dhanvanthiri Probiotics Ltd., Kodaikanal, India) using the methods described in Mangayil et al. (2021) [19]. Briefly, the SCOBY was lysed in 50 mL 1X Phosphate Buffered Saline (PBS; g/L, 8 NaCl, 0.2 KCl, 1.44 Na_2_HPO_4_, and 0.24 KH_2_PO_4_; pH 7.4) containing 1% cellulase and cycloheximide (100 mg/L), and incubated overnight (O/N) at 30 °C and 180 rpm. The cells released from SCOBY were centrifuged at 1000× *g* for 10 min at 4 °C, washed thrice with sterile PBS and serially diluted in the buffer. The presence of acetic acid bacteria was verified by plating the aliquots onto glucose-yeast extract-calcium carbonate agar (GYC; g/L, 40 glucose, 10 yeast extract, 30 CaCO_3_ and 15 agar) containing 100 g/L cycloheximide. Colonies around the CaCO_3_ solubilization zones were individually picked, streaked on Hestrin–Schramm agar (HS-glucose agar; g/L, 5 peptone, 5 yeast extract, 2.7 Na_2_HPO_4_, 1.15 citric acid and 15 agar) and incubated at 30 °C for 3–5 days. Single colonies from HS agar were inoculated in sterile 6-well culture plates (Argos Technologies, Cole-Parmer, USA) containing HS medium and the cellulose pellicles at the air/liquid interface were lysed. The cell suspensions were washed in PBS, restreaked on HS-glucose agar and the enrichment was iterated for two more rounds.

For biochemical characterization tests, the cells released from the BC pellicle, synthesized from static cultivation of the glycerol stock in HS-glucose medium, were used as the pre-inoculum. The characterization tests were conducted in MA/9 (g/L; 5.52 Na_2_HPO_4_·2H_2_O, 3.4 KH_2_PO_4_, 1 NH_4_Cl, 0.008 nitrilotriacetic acid, 1 NaCl, 0.25 MgSO_4_·7H_2_O, 0.02 CaCl_2_·2H_2_O, 0.001 FeCl_3_ and 0.2% casein amino acids) minimal medium [20] and peptone-yeast extract medium (PY; g/L, 3 peptone and 2 yeast extract) [21]. Growth only in the presence of 30% glucose, 0.35% acetic acid, 3% ethanol, or 3% ethanol with 4% acetic acid, and acetate and lactate oxidation tests, and catalase tests were conducted as described in [21]. Presence of oxidase was tested using oxidase test disks as per manufacturer’s instructions.

For strain identification by 16S rRNA gene sequencing, the genomic DNA (gDNA) was prepared, from the cells released from the BC pellicle using GeneJET Genomic DNA Purification Kit as per manufacturer’s instructions. Using the identification service offered by Macrogen (Netherlands), the 16S rRNA gene was amplified from the gDNA using primers 27F (AGAGTTTGATCMTGGCTCAG) and 1492R (TACGGYTACCTTGTTACGACTT) and sequenced with primer pairs 785F (GGATTAGATACCCTGGTA) and 907R (CCGTCAATTCMTTTRAGTTT). The 16S rRNA gene sequence can be found in the NCBI GenBank database under the accession number MT093993. Homology comparisons of the 16S rRNA gene were conducted using the nucleotide BLAST tool [22] against the NCBI GenBank 16S rRNA gene sequence repository for *Komagataeibacter* (taxid:1434011). Multiple sequence alignment and evolutionary analysis against the 16S rRNA gene sequences of *Komagataeibacter* type strains were conducted using ClustalW [23] and MEGA X using the neighbor-joining method and the Kimura 2-parameter model [24], respectively.

### 2.3. BC Production

The BC production tests were conducted in HS and MA/9 minimal medium in 6-well culture plates containing 10 mL of respective growth medium individually supplemented with 2% glucose, pure glycerol and crude glycerol statically incubated at 30 °C for 10 days. The tests were conducted in duplicate. A substrate blank (i.e., cultivation without the studied carbon sources) was included to detect the effect of yeast extract and tryptone (in HS medium) and casein amino acids (in MA/9 medium) on BC production. Additionally, non-inoculated growth medium was included as the contamination control.

BC synthesis from acetate was studied by cultivating the cells in 50 mL tubes (Star Lab, Hamburg, Germany) containing 10 mL of either HS, MA/9 and M9 (MA/9 medium devoid of casein amino acids) medium and 10 mM and 50 mM acetate. The influence of other carbon sources on BC synthesis from acetate was tested by supplementing 2% sodium gluconate or 50% of baker’s yeast hydrolysate (dry yeast purchased from local market) to MA/9 medium containing acetate. The pre-treatment of baker’s yeast was performed using the method described in [25]. The tests were conducted in duplicates in 6-well culture plates containing 10 mL of culture medium. Growth media devoid of acetate and cells were used as controls to monitor background BC production and contamination, respectively.

### 2.4. Purification of BC Pellicles and Dry Weight Measurements

The BC pellicles produced at the air/liquid interface were collected from the cultivation vessel. To eliminate the loosely bound cellulose fibrils, the BC pellicles were rinsed thoroughly with ultrapure water (Milli-Q, EMD Millipore, Darmstadt, Germany). To remove the medium components and bacterial cells entrapped within the pellicle, BC was incubated in 0.5 M NaOH solution and ultrapure water at 60 °C O/N. Following the treatments, the cellulose pellicles were washed thoroughly with ultrapure water until neutral pH was attained and oven-dried O/N on pre-weighed 46 × 46 × 8 mm weighing boats (Heathrow Scientific, Vernon Hills, IL, USA) at 60 °C. For acetate production tests in HS medium, BC pellicles were subjected to drying after collecting from the cultivation medium.

### 2.5. Amino Acid Utilization Test

For the amino acid utilization test, *K. rhaeticus* ENS9b pre-inoculum were prepared from static cultivation of cells in MA/9 medium containing 2% glucose. The pre-inoculum was inoculated to 96-well microtiter plate wells (initial OD_600nm_, 0.1) individually containing 22 L-amino acids (alanine, arginine, asparagine, aspartic acid, cysteine, cystine, glutamic acid, glutamine, glycine, histidine, hydroxyproline, isoleucine, leucine, lysine, methionine, phenylalanine, proline, serine, threonine, tryptophan, tyrosine and valine) as sole carbon source, with a final concentration of 20 mM (except for cystine and tyrosine for which the concentration was 10 mM due to low solubility) in M9 media (total volume, 200 µL/well). The tests included triplicates for each amino acid. M9 medium devoid of amino acid supplementation and *K. rhaeticus* ENS9b cells were included as experimental controls to monitor the background growth and contamination, respectively. The microtiter plates were incubated at 30 °C for 8 days and the optical density measurements at 600 nm (OD_600nm_) were taken once every 24 h.

### 2.6. Analytical Techniques

Biomass was determined as optical density measurements at 600 nm wavelength (OD_600nm_) using a spectrophotometer (Ultrospec 500 pro, Amersham Biosciences, Buckinghamshire, UK). Substrate utilization, and liquid end metabolites were analyzed using HPLC equipped with 300 mm × 8 mm Shodex SUGAR column (Phenomenex, Torrance, CA, USA), SIL-20AC HT autosampler (Shimadzu, Kyoto, Japan), RID-10A refractive index detector (Shimadzu), and 0.01 M H_2_SO_4_ as the mobile phase. The HPLC samples were prepared as described in [26] and the concentrations of carbon substrates and liquid fermentation metabolites were analyzed using respective external standards. The BC dry weights were measured using analytical balance (ES 220A, Precisa, Dietikon, Switzerland). The carbohydrate content in the baker’s yeast hydrolysate was analyzed using the phenol–sulfuric acid method described in [27] and glucose standards (0.1–1 mM).

### 2.7. Material Characterization

BC films (oven-dried prepared from MA/9 medium) synthesized from each carbon source are designated as BC—glucose, BC—glycerol, BC—crude glycerol, BC—sHS and BC—YhMA/9. BC surfaces and cross-sections were analyzed using a scanning electron microscope (SEM; Zeiss ULTRAplus, Jena, Germany).

The X-ray diffraction patterns of the dried BC films were obtained using an X-ray diffractometer (XRD; Empyrean multipurpose diffractometer, PANalytical B.V., Westborough, MA, USA) as described previously [3]. The degree of crystallinity was defined as the ratio between the sum of the 101, 10-1, and 002 peaks and the sum of the aforementioned and the amorphous peak. Peak height method (or Segal method) is the widely accepted method to calculate BC crystallinity indices (CIs) [5,28]. However, for uniformity with our previous study [3,19] and comprehensive assessment using both amorphous and crystalline regions, the CI values in this study were calculated using a peak deconvolution method [29].

The thermal behaviour of the BC films was determined using Thermogravimetric analyzer (TG 209 F3 Tarsus, Netzsch-Gerätebau GmbH, Selb, Germany). The weight loss from duplicate samples (size, 3.5–6.0 mg) was studied under N_2_ atmosphere in 30–600 °C range at a heating rate of 10 °C/min.

### 2.8. Genome Sequencing, Assembly and Bioinformatics

*K. rhaeticus* ENS9b gDNA was extracted as mentioned previously (section Isolation, culturing and characterization of BC-producing isolates) and sequenced at Novogene Europe (Cambridge, UK) using Illumina Novaseq 6000. The raw reads were trimmed using Trimmomatic [30] and de novo assembled into contigs by SPAdes [31]. The contig scaffolding was conducted by SOPRA scaffold assembly tool at Galaxy Europe web platform (usegalaxy.eu; Galaxy Version 0.1) using the SPAdes contig assembly and the paired-end Illumina sequencing data [32]. The minimum contig length used in scaffold assembly was assigned as 200 bp. Misassemblies were identified using QUAST (5.0.2) [33]. Contig reordering, misassembly correction and gap filling were conducted using GFinisher with *K. rhaeticus* iGEM genome (GenBank accession number, LT575493.1) as the reference sequence [34] and the genome quality was assessed again using QUAST. The genome was annotated using Prokka [35]. The genome completeness was analyzed using the CheckM plugin in the KBase web platform [36,37]. Functional annotation was conducted through the KEGG Automatic Annotation Server (https://www.genome.jp/kaas-bin/kaas_main, accessed on 10 September 2020) [38]. Proteins associated with cellulose synthesis, substrate catabolism, and gluconeogenesis were identified either through manual search within the Prokka annotated GenBank file using Unipro UGENE software (v. 33.0) or from KEGG orthology and the generated pathways [39]. For functional analysis of the annotated proteins and domain predictions, the query amino acid sequences were searched against NCBI’s conserved domain database (CDD) and InterProScan signatures [40,41]. Plasmids in the raw sequencing reads and the assembled contigs were identified using plasmidSPAdes [42] and Recycler, respectively [43]. Genome coverage was estimated using Bowtie2 [44]. The origin of replication (oriC) was predicted using the Ori-finder tool (http://tubic.org/Ori-Finder/, accessed on 5 September 2020) [45]. For rRNAs and tRNA prediction, a homology search was conducted using RNAmmer [46] and tRNAscan-SE [47] tools at the RNAspace (v1.2.1, rnaspace.org, accessed on 28 September 2020) web environment [48].

The chromosome sequence of *K. rhaeticus* ENS9b can be found in the NCBI GenBank under the accession number CP061369 and the plasmids at CP061370 (p9b_1), CP061371 (p9b_2), CP061372 (p9b_3), CP061373 (p9b_4) and CP061374 (p9b_5).

## 3. Results

### 3.1. Characterization and Classification of the Cellulose-Producing Isolate

Colony isolation from CaCO_3_ solubilized zones and iterated streaking of cells from BC pellicles on HS-glucose agar resulted in the enrichment of small, irregularly edged, white-colored smooth colonies (Figure S1A). Prolonged incubation resulted in the appearance of a white mass on the colony tip changing the morphology to pulvinated colonies which were difficult to pick using an inoculation loop. The isolate, hereafter designated at ENS9b, appeared as rod-shaped cells either singularly, in pairs or in chains with a cell size of 2.6–4.5 µm × 0.6–0.7 µm (Figure S1B).

**Figure 1:**
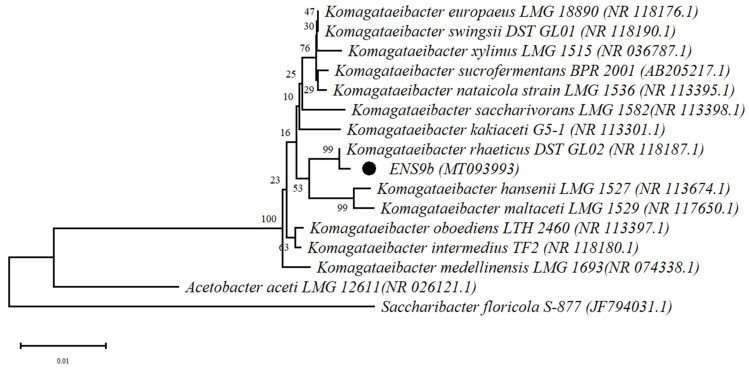
Phylogenetic relationship of ENS9b and *Komagataeibacter* type strains. The 16S rRNA gene tree was rooted using *Saccharibacter floricola* S-877^T^ and *Acetobacter aceti* LMG 12611^T^. The position of ENS9b in the phylogenetic tree is highlighted with a dot. NCBI accession numbers are provided in parenthesis.

Phylogenetic analysis using the ENS9b 16S rRNA gene against the *Komagataeibacter* type strains positioned the isolate among *K. rhaeticus* showed a 99% similarity towards *K. rhaeticus* DST GL02^T^ (Figure 1).

To restrict the influence of peptone and yeast extract on the isolate’s growth, the biochemical characterization tests were initially conducted in MA/9 minimal medium. However, similar to that observed from a related species [19], the isolate grew well with 30% glucose but poorly in MA/9 medium containing 0.35% acetic acid, 3% ethanol, and 3% ethanol and 4% acetic acid. Thus, the characterization tests were conducted in PY medium [21]. For cultivations containing 3% ethanol and 4% acetic acid, the medium pH remained stable until day 11, with a slight drop thereafter (Figure S2). Growth in the presence of 0.35% acetic acid did not reveal any major changes in the medium pH trend (Figure S2). However, the capacity of ENS9b to metabolize acetic acid was confirmed by the formation of BC pellicles and its absence in the control cultivation (PY medium without 0.35% acetic acid or 3% ethanol, Figure S3). Similar to other *Komagataeibacter* spp., *K. rhaeticus* ENS9b showed positive and negative results for catalase and oxidase tests, respectively; did not require acetic acid for growth, oxidized acetate, and lactate in the PY medium and demonstrated acetic acid overoxidation (Figure S4) [49,50].

### 3.2. Bacterial Cellulose Production in Rich and Minimal Growth Media

**Figure 2:**
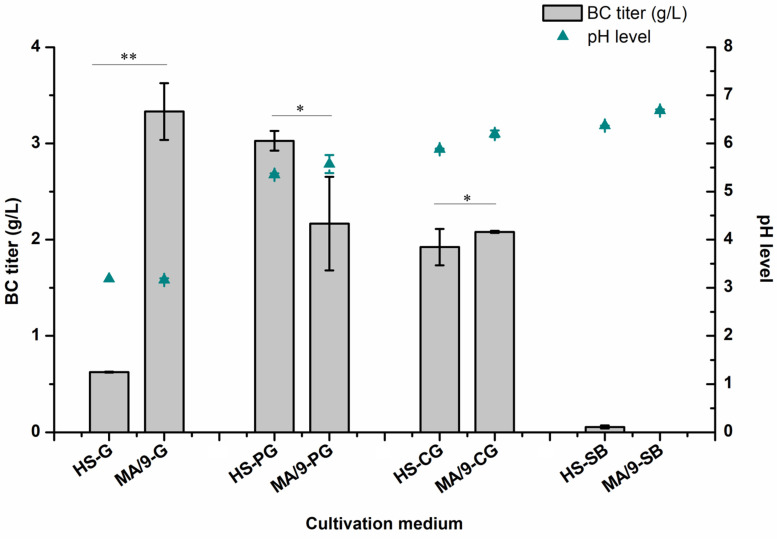
Bacterial cellulose (BC) production (bars), and pH (line) plots from static cultivations in HS and MA/9 growth media supplemented with 2% glucose (G), pure glycerol (PG) and crude glycerol (CG). Substrate blank (SB) was included to identify the contribution of medium components (yeast extract and peptone and casein amino acids in HS medium and MA/9 medium, respectively) towards BC production. The pH levels of the medium after the cultivation period are presented as scattered plots (Cyan triangles). The data points represent the mean experimental results and standard deviations from duplicate cultivations. In some cases, the error bars are smaller than the symbol. Statistical differences between the results of BC production in HS and MA/9 media containing similar growth substrate were analyzed using Two sample t-test with equal variances. Samples with *p* > 0.05 and *p* < 0.05 are represented by * and **, respectively.

The BC production and pH profiles from cultivations in the HS and MA/9 media with respective carbon sources are presented in Figure 2. When grown in the HS medium, *K. rhaeticus* ENS9b produced the highest BC titer (and yield) in the medium containing pure glycerol (3.0 ± 0.1 g/L and 1.5 mg/g_substrate_), utilizing 55 ± 0.5% of the substrate. With crude glycerol as the substrate, the strain utilized 48% of the carbon source, producing 1.9 ± 0.2 g/L BC (0.9 mg/g_substrate_). After the 10-day cultivation period, *K. rhaeticus* ENS9b completely utilized the supplemented glucose albeit producing only 0.6 ± 0.0 g/L BC and gluconic acid as the main liquid end-metabolite (14.5 ± 0.3 g/L, corresponding to 74% of the initial glucose concentration). Nevertheless, the HS medium containing other carbon compounds, such as yeast extract and tryptone, can contribute towards biomass and background BC production [15,19]. In substrate blank cultivation, *K. rhaeticus* ENS9b produced 0.05 ± 0.0 g/L BC.

In contrast to the HS medium, BC production was not observed from cultivations in the MA/9 medium devoid of studied carbon sources (substrate blank). Growth in the MA/9-glucose medium improved BC synthesis (3.3 ± 0.3 g/L and 1.7 mg/g_substrate_) and reduced gluconate generation (7.2 ± 0.2 g/L, corresponding to 35% of initial substrate concentration). With pure and crude glycerol as the sole carbon sources, *K. rhaeticus* ENS9b utilized 72% of the supplemented substrate synthesizing 2.2 ± 0.1 and 2.1 ± 0.1 g/L of BC (~1.0 mg BC/g_substrate_), respectively.

### 3.3. BC Film Characterization

**Figure 3:**
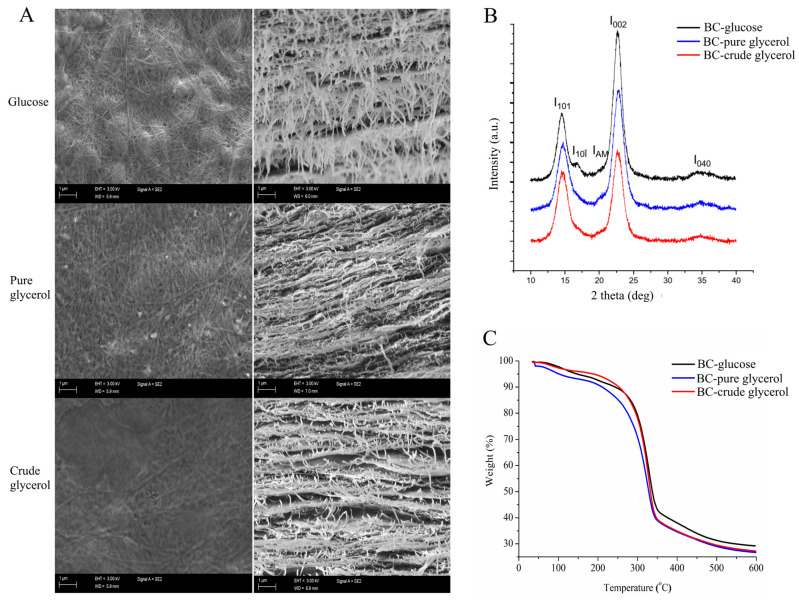
BC film characterization. (**A**) Scanning electron microscopy (SEM) surface and cross-sectional images, (**B**) X-ray diffraction (XRD) diffractograms and (**C**) thermogravimetric analysis (TGA) of BC films synthesized from MA/9 medium containing 2% glucose (BC—glucose), pure glycerol (BC—pure glycerol) and crude glycerol (BC—crude glycerol). The diffraction peaks subfigure (**B**) at I101 and I10ī, I002 and I040, and IAM represents the crystalline Iα, Iβ and amorphous regions.

The surface and cross-sections as well as the results of XRD and thermogravimetric analyses (TGA) of BC films synthesized by *K. rhaeticus* ENS9b grown in the MA/9 medium with 2% glucose, pure glycerol and crude glycerol, are shown in Figure 3. The surface and cross-sections of BC synthesized from different carbon sources (Figure 3A) demonstrated the cellulose fibrils to contain the general crisscross network with layered ordered interconnected structures consisting of nanocellulose fibrils entangled within the BC pellicles.

The XRD analysis of BC films synthesized by *K. rhaeticus* ENS9b grown in the MA/9 medium with 2% glucose, pure glycerol and crude glycerol are shown in Figure 3B. Consistent with previous reports [19,51], the diffractograms revealed two dominant peaks between 14.6° and 17.0°, and between 22.8° and 25.1° representing the cellulose I allomorphs Iα and Iβ, respectively. Though the peaks have low intensities and asymmetric shapes, amorphous regions in BC—glucose, BC—pure glycerol and BC—crude glycerol were identified at peaks 21.6°, 21.1° and 20.3°, respectively. The CI values of BC films produced from glucose, and pure and crude glycerol were 87 ± 13%, 89 ± 10% and 96 ± 2%, respectively.

The results from thermogravimetric analysis (TGA) of BC films are presented in Figure 3C. The mass losses were observed in three stages. At the first stage (30–120 °C), a primary mass loss due to moisture removal was observed as 5%, 5% and 4% for BC—glucose, BC—pure glycerol and BC—crude glycerol, respectively. The second phase, i.e., destruction of crystalline regions and decomposition of the cellulose into glucopyranose monomers, leading to a sharp weight loss, was observed at temperatures between 280 °C and 300 °C. During this phase, BC—glucose, BC—pure glycerol and BC—crude glycerol demonstrated a major mass loss of 59%, 64% and 69%, respectively. Though the mass loss values varied slightly, the residual mass (%) of the tested BC films remained in the ranges of 27–29%.

### 3.4. Genome Features of K. rhaeticus ENS9b

**Figure 4:**
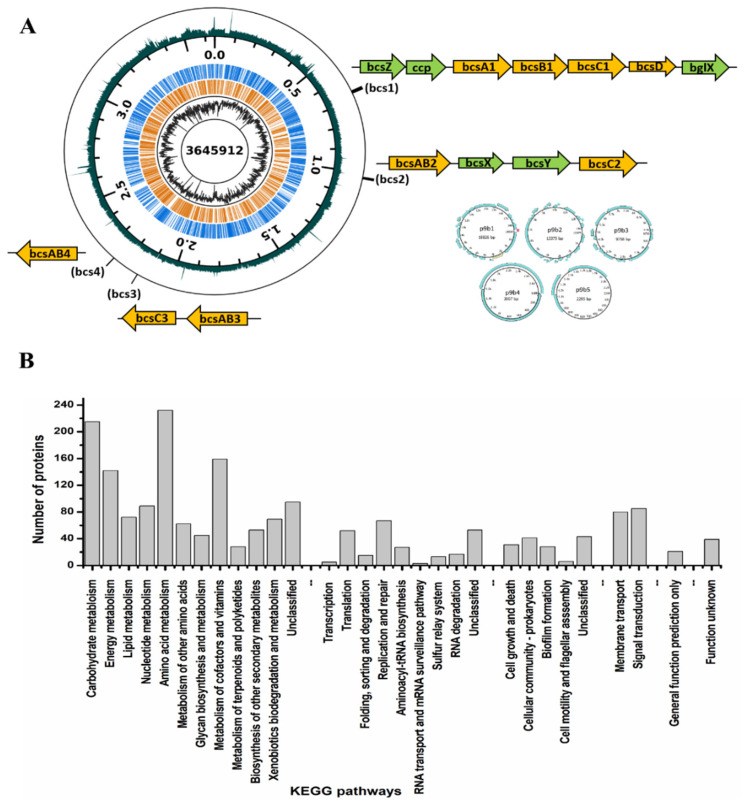
Overview of *K. rhaeticus* ENS9b genome. (**A**) Genome map. The *K. rhaeticus* ENS9b genome totals 3.69 Mbp (GC% of 63.05%), containing 3360 protein-coding gene predictions, 3 rRNAs, 1 tmRNA, 46 tRNAs and 3 non coding RNAs. A genome completeness of 100% was identified from CheckM. The genome consists of a chromosome of 3.65 Mbp (3302 predicted protein-coding regions). At least three putative plasmids; p9b1 (18.8 Kbp), p9b2 (13.4 Kbp), p9b3 (9.8 Kbp), were identified in *K. rhaeticus* ENS9b genome ((**A**) inset). Although the replicon could not be determined, as similarly reported for other *K. rhaeticus* isolates [7,19], two putative plasmid sequences of sizes 3 Kbp (p9b4) and 2.3 Kbp (p9b5) were detected by plasmidSPAdes. Seven contigs (totaling 90 Kbp) could not be confidently placed in the genome. The displayed data from center to perimeter are: chromosome size (bp), GC-percentage, CDSs on the reverse strand (in the middle, orange), CDSs on the forward strand (in the middle, blue), chromosome position (major ticks 500 kbp, minor ticks 100 kbp), and coverage (outermost). Both GC-percentage and coverage were calculated with 1 kbp window size. GC-percentage ranged from 33.6% to 75.5% and coverage (number of reads mapping to the locus) from 380 to 49600. Coverage is displayed with logarithmic scaling. The bcs operon (orange filled arrows) and the flanking accessory genes (green filled arrows) indicated in the manually curated figures directs to their position in the genome. (**B**) KEGG classification and functional categorization.

The assembly statistics and general features of the *K. rhaeticus* ENS9b genome are presented in Table S1. The genome map and functional categorization of the gene annotations to KEGG orthology are presented in Figure 4. According to the functional analysis (KEGG classifications and InterProScan), manual search and amino acid alignments (BlastP), and domain predictions (CDD), genes encoding for proteins involved in carbohydrate uptake and metabolism, bacterial cellulose synthesis, and gluconeogenesis from the central metabolic pathway were identified from the genome. The genomic positions of genes encoding for the aforementioned enzymes and the respective amino acid sequences are presented, in order, in Supplementary Material.

#### 3.4.1. BC Biogenesis Machinery

The *K. rhaeticus* ENS genome contained four copies of the bcs operon. The complete bcs operon (bcs1, 9100 bp) comprising individual physically adjacent bcsA1, bcsB1, bcsC1 and bcsD genes, in order, was identified at genomic position 666919:676019 bp. The gene cluster was flanked by accessory genes bcsZ (genomic position 664722:665759 bp), ccpAx (genomic position 665756:666736 bp), and bglX (genomic position 676251:678452 bp) encoding for β-1,4-glucanase, cellulose complementing factor protein, and β-glucosidase, respectively. An additional copy of the bglX gene (bglX2) was identified at genomic position 522700:524739 bp. A second bcs operon (bcs2) comprising a gene fusion encoding for the catalytic and regulatory subunits, bcsAB2, and bcsC2 genes was identified at genomic position 1020008:1030553 bp. The third (at genomic position 2136184:2144443 bp) and fourth (at genomic position 2248494:2253065 bp) bcs clusters were arranged in complement in the genome and comprised the bcsAB3 and bcsC3 genes, and the bcsAB4 genes, respectively. These bcs clusters were not flanked with accessory genes associated with BC assembly/production.

#### 3.4.2. Predicted Genes Involved in Carbohydrate Uptake and Metabolism

In the *K. rhaeticus* ENS9b genome annotations, seven genes that encode for putative carbohydrate-selective porins of the porin B (OprB) family were identified. Additionally, three copies of aquaporin Z, a major bacterial protein involved in water and glycerol diffusion across the cell membrane were identified in the genome. Similar to other *Komagataeibacter* spp., an incomplete glycolytic pathway due to the lack of phosphofructokinase was observed from the *K. rhaeticus* ENS9b genome [52,53]. Glucose oxidation occurs through the pentose phosphate pathway (PPP) and Entner–Duodoroff pathway (EDP) via gluconate as the intermediate (catalysed by the two quinoprotein glucose dehydrogenases genes (*gdh*) found at genomic positions of 1924182:1926572 and 2594170:2596791 bp). For glycerol metabolism, genes encoding for glycerol uptake facilitator protein (at positions 1285133:1285972 bp), glycerol kinases (1286008:1287507 bp and 1389048:1390547 bp), glycerol dehydrogenases (1201435:1203437 and 1673449:1676036 bp), dihydroxyacetone kinase (1848749:1850380 bp) and triosephosphate isomerase (2116868:2117611 bp) entering into the gluconeogenetic, and central metabolic pathways were identified in the genome.

#### 3.4.3. Putative Genes Encoding for Enzymes Involved in Redirecting Carbon from the Krebs Cycle

Indications of BC biogenesis from acetate and putative genes responsible for redirecting the carbon from the Krebs cycle towards gluconeogenesis in *K. rhaeticus* isolate were identified in our previous study [19]. Similar to our finding, the *K. rhaeticus* ENS9b genome lacked the genes encoding pyruvate synthase (acetyl-CoA to pyruvate) and phosphoenolpyruvate carboxykinase (oxaloacetic acid to phosphoenolpyruvate). However, the genome contained annotations for genes encoding NAD-dependent malic enzyme (catalyzing the reversible malate to pyruvate reaction) and pyruvate phosphate dikinase (pyruvate to phosphoenol pyruvate).

### 3.5. BC Production from Acetate

To validate the catalytic activities of in silico predicted enzymes involved in gluconeogenesis and corroborate growth and BC production from acetyl-CoA, the central compound linking the Krebs and gluconeogenetic pathways, *K. rhaeticus* ENS9b was grown in the presence of acetate.

Acetate utilization and BC production was tested in the PY, HS, MA/9 and M9 media containing 10 mM and 50 mM acetate (Table 1). In PY medium, BC, observed as cellulose fibrils, was produced in both control (devoid of acetate) and sample cultivations. However, due to the absence of an intact pellicle, the fibrils could not be effectively separated from the culture medium for quantification (Figure S5). At the end of the cultivation in the PY-10 mM medium, the strain utilized 78 ± 5% acetate. However, growth in the PY-50 mM medium increased acetate concentration (61 ± 7 mM). In the control cultivation without acetate, the strain produced 3.5 mM acetate.

**Figure 5:**
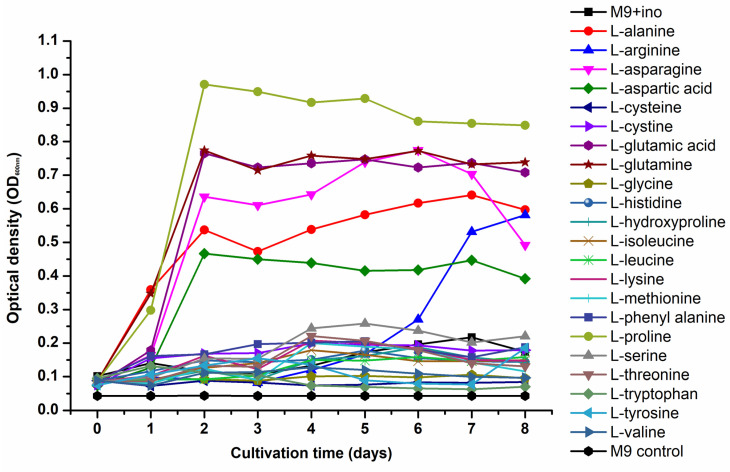
Amino acid utilization profile of *K. rhaeticus* ENS9b cells grown in M9 medium individually containing 22 L-amino acids. The test was conducted in 96-well microtiter plate wells containing a total volume of 200 µL/well. The experimental controls included M9 medium containing cells but devoid of amino acid supplementation (M9+ino control) and M9 medium devoid of cells and amino acids (M9 control). The microtiter plates were incubated at 30 °C for 8 days and the OD_600nm_ measurements were taken once every 24 h. The presented data is the averaged value obtained from triplicate cultivations. The error bars are not included for clarity.

After 14 days of static incubation in HS medium without acetate supplementation, *K. rhaeticus* ENS9b produced 7 mg of fragile BC pellicles (Figure S6). As the pellicles were fragile, they were not subjected to alkali and MQ washes, and were oven-dried soon after being collected from the cultivation vessel. Supplementation of 10 mM and 50 mM acetate to the HS medium resulted in intact pellicles. In the HS medium containing 10 mM and 50 mM acetate, the *K. rhaeticus* cells synthesized 17 ± 4.5 mg and 20 ± 0.9 mg BC, respectively (Table 1). The BC pellicle weights from each replicate cultivation are presented in Figure S6C. In the M9 medium containing 10 mM and 50 mM acetate, cell growth was observed without BC synthesis (Table 1). Similar to the M9 medium, BC synthesis was not observed in MA/9 cultivations. Nevertheless, supplementation of casein amino acids in the MA/9 medium resulted in a ~2- to 9-fold increase in OD_600nm_ values. An amino acid utilization test was conducted to study the influence of individual L-amino acids on *K. rhaeticus* growth in the M9 medium. The cells could utilize L-alanine, L-arginine, L-asparagine, L-aspartic acid, L-glutamine, L-glutamic acid and L-proline as sole carbon sources for biomass formation (Figure 5).

**Figure 6:**
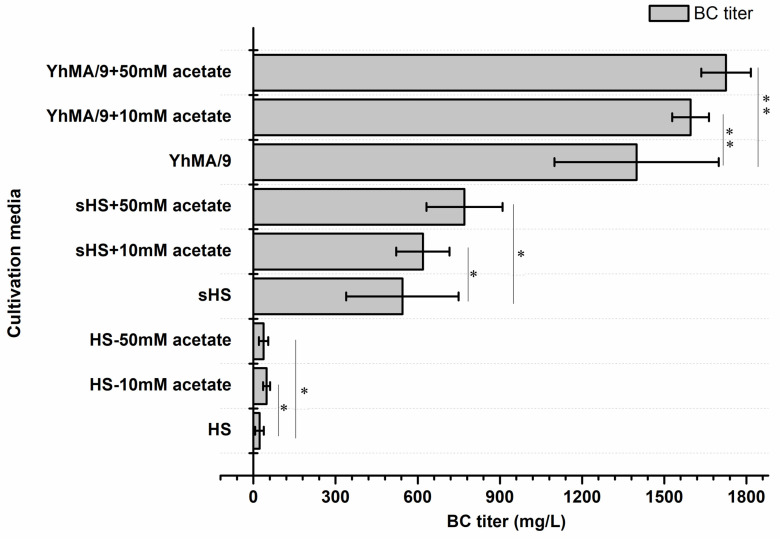
BC production of *K. rhaeticus* ENS9b cells in control and sample cultivations containing 10 mM and 50 mM acetate in HS, sHS and YhMA/9 media. The presented results are the averaged values (duplicates for control cultivations and triplicates for media with acetate) and the error bars represent the standard deviations. A two sample *t*-test with equal variances using the log10 transformed values was implemented to compare the control and sample cultivations. Samples with *p* > 0.05 and *p* < 0.05 are represented by * and **, respectively.

In comparison to the control cultivation, an improvement in BC production was observed from *K. rhaeticus* ENS9b cells grown in the HS medium containing acetate. However, in a medium devoid of readily metabolizable substrates the carbon in acetate was directed towards biomass formation (Table 1). Taking these cues, we studied the effect of baker’s yeast hydrolysate supplementation in the MA/9 medium (YhMA/9) on BC production from acetate [25]. HS and simulated HS media (sHS), i.e., HS medium with 50% of baker’s yeast hydrolysate and 0.2% casein amino acids to replace the yeast extract and casein amino acids, respectively, were used as the experimental control (Figure S7A). Figure 6 presents the BC titers from acetate supplementation to HS, sHS and YhMA/9 media. In the cultivation media devoid of acetate, *K. rhaeticus* ENS9b cells synthesized BC from the control cultivations. In comparison to the HS medium, the BC production was higher in sHS and YhMA/9 media, attributed to the inclusion of 50% baker’s yeast hydrolysate. The phenol-sulfuric acid test indicated a total carbohydrate content in HS (contributed by yeast extract) and sHS (from 50% of baker’s yeast hydrolysate) media as 7.7 mM and 76 mM, respectively. The results presented in Figure 6 clearly show the improvement in BC production from acetate supplemented media. However, only the YhMA/9–acetate media cultivations demonstrated statistically significant (*p* < 0.05, two-sample *t*-test) titers. Deducting the contribution of yeast extract and baker’s yeast hydrolysate in respective media, BC titers of 15–25 mg/L, 75–227 mg/L and 197–327 mg/L were obtained from acetate supplemented HS, sHS and YhMA/9 media, respectively.

SEM, XRD and TGA characterization of BC films produced from acetate-supplemented sHS and YhMA/9 media are shown in Figure S8. Consistent with the prior results (section BC film characterization), peaks representing cellulose I allomorphs (Iα and Iβ) and amorphous regions were identified at 14.7°, 16.9° and 22.2°, respectively. The peak deconvolution study identified the CI values from BC—sHS and BC—YhMA/9 as 94% and 92%, respectively. TGA analysis indicates a similar mass loss trend among the BC films, with a residual mass of ~28%.

## 4. Discussion

BC is a versatile biopolymer synthesized by bacteria that require mild conditions, simple growth media, and can utilize wide ranges of substrates for growth. *Komagataeibacter* strains have been investigated for their growth and BC production capacities from conventional sugars, industrial wastes and detoxified lignocellulosic biomass [9,13,19,54,55,56,57]. Glucose is an excellent carbon source for BC production, as it is easily transported into the cell and is efficiently incorporated into the cellulose biosynthetic pathway. However, *Komagataeibacter* spp. lacks phosphofructokinase to complete the glycolytic pathway. Glucose oxidation in *Komagataeibacter* spp. occurs in two routes, via glucose-6-phosphate (entering into PPP) and through gluconate generation (which is exported extracellularly or directly oxidized into the medium by surface-exposed gdh). In addition to the drop in the medium pH, gluconate synthesis reduces the glucose availability for BC biosynthesis [3,8,19]. Compared to the HS medium, the improved production titer (3.3 ± 0.3 g/L BC) and reduced gluconic acid generation (7.2 ± 0.2 g/L) from MA/9 cultivations can be attributed to the buffering capacity in the growth medium [58]. Additionally, the presence of essential macro and micronutrients in the original MA/9 medium composition, may positively contribute to bacterial growth and cellular metabolism [59]. The obtained BC titers from the MA/9 medium are comparable to the previously published results from minimal media. For instance, Forng et al. (1989) reported a modest BC titer of 0.1 g/L from *A. xylinum* static cultivations [17]. In another study, de Souza et al. (2019) reported a titer of 0.22 g BC from *K. hansenii* ATCC 23769 grown in 10 mL of minimal medium containing glucose [59]. Wu et al. (2019) reported a BC titer of ~1.5 g/L from *A. xylinum* using crude glycerol, a by-product generated from biodiesel synthesis using kitchen waste [9]. Recently, using a *Komagataeibacter* isolate cultivated in similar conditions and 20 g/L glucose as the carbon source we reported a titer of 2.2 ± 0.1 g/L BC [19]. In contrast to glucose cultivations in this study, glycerol as the growth substrate demonstrated an improved substrate utilization (HS medium, 55 ± 0.5% and MA/9 medium, 72 ± 4%), albeit with a slight drop in the BC production titer (HS medium, 3.0 ± 0.1 g/L; MA/9 medium, 2.2 ± 0.1 g/L BC). This could be attributed to the functional activities of enzymes involved in BC biogenesis and gluconeogenesis. BC synthesis is regulated by the enzymatic activities of diguanylate cyclase, phosphodiesterase, UDP-glucose pyrophosphorylase, and bcs operon, which are regulated by the physiological conditions in which the bacterium is cultivated [60]. When *Komagataeibacter* spp. are grown in a minimal medium, the rate-limiting step for BC biogenesis is partly determined by the carbon source of choice. In the case of glycerol, reduced carbon flux towards the gluconeogenesis route could be attributed to the production metrics [61].

From an industrial point of view, it is reported that a BC titer of 15 g/L in 50 h (productivity, 0.3 g/L/h) is required to match the production efficiency of plants [62]. Thus far, the highest volumetric yield (15 g/L BC in 7 days; productivity 0.1 g/L/h) has been reported from a *Komagataeibacter* isolate cultivated in HS medium containing 4% glucose and 1.4% ethanol as the inducer [63]. However, utilization of conventional sugars and inducing compounds hinders the production costs and potential for scale-up. Integrating waste valorization with cellulose production is one way to upscale the production processes. Agricultural and industrial waste residues, to name a few, citrus pulp waste, distillery wastewater, crude glycerol, fermentation by-products and detoxified lignocellulosic hydrolysates have been employed as alternative carbon and nitrogen sources for BC production, resulting in titers ranging from 1–13 g/L in 2–15 cultivation days [13,19,64,65,66]. A recent study reported the production of 12–16 g/L BC from lignocellulosic biomass hydrolysates using *K. xylinus* ATCC 53524 statically grown in a medium containing 50 g/L glucose and 20 g/L corn steep liquor. The study by Guo et al. (2013) reported that medium detoxification was an important pretreatment step while utilizing lignocellulosic biomass for BC synthesis, as the BC producers are sensitive towards the LDMs present in the hydrolysates [11]. Nevertheless, their subsequent study revealed the capacity of K. xylinus cells in the biotransformation of model lignin monomers, albeit in low concentrations, and in detoxifying lignocellulosic hydrolysates [67]. In the study, the authors reported BC titers of 3.4 g/L, 6.6 g/L, 2.9 g/L, and 6.2 g/L from a medium containing glucose and coniferyl aldehyde (1.5 mM), ferulic acid (2 mM), vanillin (2.5 mM) and 4-hydroxybenzoic acid (2.5 mM) supplementations, respectively. The identification of respective enzymes involved in LDM biotransformation could pave the way to exciting biotechnological prospects. Nevertheless, for BC synthesis from LDM catabolism, carbon needs to be redirected from acetyl-coA towards gluconeogenesis. Initial observations of BC formation from acetate were observed from biochemical characterization tests. In *Komagataeibacter* spp., acetyl-coA synthetase converts acetate to acetyl-coA, thereby entering into the Krebs cycle. Similarly, in other *K. rhaeticus* isolate and *Escherichia coli* genomes found, the annotations for genes encoding NAD-dependent malic enzyme and pyruvate phosphate dikinase in the *K. rhaeticus* ENS9b genome allowed us to hypothesize that the carbon from Krebs cycle could be routed towards gluconeogenesis [19,68]. In this proof-of-concept study, the hypothesis was validated using acetate supplementation to the growth medium. Acetate is generally used as an inducer compound to augment BC production [55,58]. Wang et al. (2018) reported that *Komagataeibacter* sp. W1 strain could synthesize fragile BC pellicles (~70 mg) from HS medium containing 20 g/L of acetate [55]. Our tests identified that the availability of other carbon compounds was crucial to promote biomass formation and generate thick BC pellicles. In the absence of readily utilizable carbon (MA/9 and M9 media), acetate was primarily utilized for biomass formation (Table 1). In comparison to the cultivations in M9 medium containing acetate, a ~2- to 9-fold biomass increase (OD_600nm_) observed from acetate supplemented MA/9 medium validates the bacterium’s capacity to utilize the casein amino acids for cell growth (Table 1, Figure 5). The observation of fragile BC pellicles synthesized from an acetate-supplemented HS medium in this study, corroborates to the result from Wang et al.’s (2018) study, suggesting that the yeast extract in the HS medium can provide the carbon for BC synthesis. Taking these observations, we hypothesized that supplementing the growth medium containing acetate with other readily utilizable carbon may be beneficial in supporting the bacterium for both biomass formation and BC synthesis. BC biogenesis in YhMA/9 and sHS medium confirmed the hypothesis, suggesting an efficient route for both waste valorization and BC production from acetate (Table 1, Figure 6). Furthermore, the ~2- to 3-fold increase in the BC titers synthesized from YhMA/9 medium, compared to the titers obtained from sHS medium, verifies the impact of the cultivation media. Future work will investigate the applicability of spent yeast hydrolysates on BC synthesis, bioprocess optimization using statistical design models and adapted laboratory evolution of the *K. rhaeticus* strain on LDMs.

## Figures and Tables

**Table 1:** Biomass, BC production and acetate utilization from static growth in PY, HS, MA/9 and M9 media containing 10 mM and 50 mM acetate.

Growth Medium	Carbon-Containing Compounds	BC Production	OD_600nm_	Endpoint Acetate Concentration (mM) and Percentage Utilized (%)
PY	2 g/L YE	Very loose pellicles. Could not be quantified.	1.4	3.5 mM
	2 g/L YE + 10 mM acetate	Loose pellicles. Could not be quantified.	1.5 ± 0.2	1.9 ± 0.7 (78 ± 5.4%)
	2 g/L YE + 50 mM acetate	Loose pellicles. Could not be quantified.	1.8 ± 0.4	61.2 ± 7.8 (Higher than initially supplemented)
HS ^1^	5 g/L YE	7 mg	Cells within the pellicle. Did not quantify.	ND
	5 g/L YE + 10 mM acetate	17 ± 4.5 mg	Cells within the pellicle. Did not quantify.	2.5 ± 2.0 (74 ± 29.4%)
	5 g/L YE + 50 mM acetate	20 ± 0.9 mg	Cells within the pellicle. Did not quantify.	34.6 ± 4.9 (33 ± 9.4%)
MA/9	-	ND	0.8	ND
	10 mM acetate	ND	1.4 ± 0.2	0.9 ± 0.0 (91.5 ± 1.5%)
	50-mM acetate	ND	1.5 ± 0.1	7.5 ± 3.5 (79 ± 7.8%)
M9	-	ND	0.1	ND
	10 mM acetate	ND	0.5 ± 0.1	0.4 ± 0.0 (95.3 ± 0.2%)
	50 mM acetate	ND	0.9 ± 0.1	2.2 ± 1.5 (98.2 ± 3.6%)

^1^ Due to the fragile nature of the BC pellicles produced from the control cultivation, the pellicles were not subjected to alkali and MQ wash. The pellicles from the cultivation vessel were collected and oven-dried at 60 °C. For comparison, the pellicles synthesized from HS containing 10 mM and 50 mM acetate were treated similarly. These pellicles can contain medium components that may alter the titers. Thus, the dried weights of untreated pellicles are presented.

## Data Availability

The data presented in this study are available in the article or Supplementary Materials.

## Supplementary Materials

The following are available online at https://www.mdpi.com/article/10.3390/microorganisms9112230/s1, Figure S1. (A) Colony and (B) cell morphologies of ENS9b. The images were cropped, and brightness and contrast were adjusted to improve clarity. Figure S2. pH changes during cultivations in MA/9 containing 30% glucose and PY medium containing 3% ethanol, 0.35% acetic acid, 3% ethanol and 4% acetic acid. The cultivations were conducted at 30 °C and 230 rpm for 14 days. Averaged values and standard deviations from duplicate cultivations are presented. In some cases, the error bars are smaller than the symbol. Figure S3. BC pellicle formation in PY medium containing 0.35% acetic acid, 3% ethanol and 4% acetic acid (A). Verification of BC pellicle, produced after 14-day cultivation in PY medium individually containing the above-mentioned carbons sources, were conducted using 1% cellulase. Lysis of BC pellicle and release of cells after the cellulase treatment is shown in (B). The images were cropped, and brightness and contrast were adjusted to improve clarity. Figure S4. Overoxidation of acetic acid by ENS9B. Single colonies were streaked onto PY medium containing 2% ethanol and 0.0002% bromothymol blue as the pH indicator. Figures (from left to right) shows the acidification (blue to yellow coloration) and neutralization (yellow to blue) of PY agar during 2, 6, 10 and 20 days of incubation at 30 °C. The images were cropped, and brightness and contrast were adjusted to improve clarity. Figure S5. BC pellicle production by *K. rhaeticus* ENS9b cells statically grown in 50-mL tubes containing 10 mL (A) PY substrate control (i.e., devoid of acetate), (B) PY medium supplemented with 10-mM acetate and (C) PY medium supplemented with 50-mM acetate for 14 days at 30 °C. (D) and (E) are the top-view images of sub-figures (B) and (C), respectively, showing the cellulose pellicles. The images were cropped, and brightness and contrast were adjusted to improve clarity. Figure S6. BC production by *K. rhaeticus* ENS9b cells grown statically in HS control (HS, without acetate) and sample (A10 and A50, medium containing 10-mM and 50-mM acetate) cultivation medium. (A) and (B) presents the pristine BC pellicles and the fragile pellicles produced from the control cultivation, respectively, placed on 46 × 46 × 8 mm pre-weighed weighing boats. The images were cropped, and brightness and contrast were adjusted to improve clarity. (C) BC pellicle weights produced from HS medium with and without acetate supplementation. The BC pellicles produced from respective cultivations were collected and dried O/N at 60 °C. The graph presents the BC weights (in milligrams) obtained from each replicate. The pellicles were not treated with alkali and MQ. Thus, the medium components present in the pellicles may alter the titers and thus, the BC weights are presented. Figure S7. Dried BC sheets. BC pellicles produced by *K. rhaeticus* ENS9b grown statistically in (A) control cultivations devoid of acetate, (B) HS medium, (C) sHS medium and (D) YhMA/9 containing 10-mM and 50-mM acetate. The BC pellicles washed with alkali and ultra-pure water were placed on 46 × 46 × 8 mm pre-weighed weighing boats and dried O/N at 60 °C. The images were cropped, and brightness and contrast were adjusted to improve clarity. Figure S8. BC film characterization. (A) TGA (B) XRD diffractograms and (C) SEM analysis of BC films synthesized from acetate supplemented sHS, YhMA/9 medium and respective control cultivations devoid of acetate (ENS9b_YhMA/9 and ENS9b_sHS). Table S1. General genome and assembly statistics of *K. rhaeticus* ENS9b genome. File S1. Amino acid sequences.
