# Supplementary material for: Characterization of Komagataeibacter Isolate Reveals New Prospects in Waste Stream Valorization for Bacterial Cellulose Production
Source: Microorganisms. 2021 Oct 26;9(11):2230. doi: 10.3390/microorganisms9112230 (PMC8621423; doi:10.3390/microorganisms9112230)
Supplement: Supplementary file 1 [file microorganisms-09-02230-s001.zip › microorganisms-1360239-supplementary.pdf]

Characterization of *Komagataeibacter* isolate reveals new prospects in waste stream valorization for bacterial cellulose production

Pietro Cannazza [1,*], Antti J Rissanen [2], Dieval Guizelini [3], Pauli Losoi [2], Essi Sarlin [2], Diego Romano [1], Ville Santala [2] and Rahul Mangayil [2,*]

[1] Department of Food, Environmental and Nutritional Sciences (DeFENS), University of Milan, Via Celoria 2, 20133 Milan, Italy

[2] Faculty of Engineering and Natural Sciences, Tampere University, Tampere, Finland

3 Graduate Program in Bioinformatics, Sector of Professional and Technological Education, Federal University of Parana (UFPR), Curitiba, PR, Brazil

Corresponding Authors:

Rahul Mangayil

Korkeakoulunkatu 6, Tampere, 33720, Finland

Email address: rahul.mangayil@tuni.fi

Pietro Cannazza

Via Celoria 2, Milan, 20133, Italy

Pietro.cannazza@unimi.it

Supplementary figures

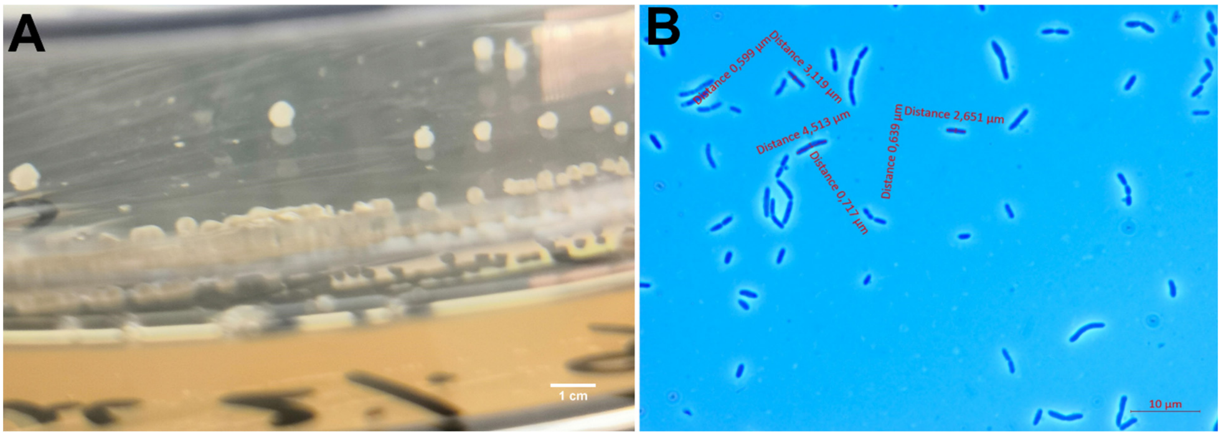

Figure S1: (A) Colony and (B) cell morphologies of ENS9b. The images were cropped and brightness and contrast were adjusted to improve clarity

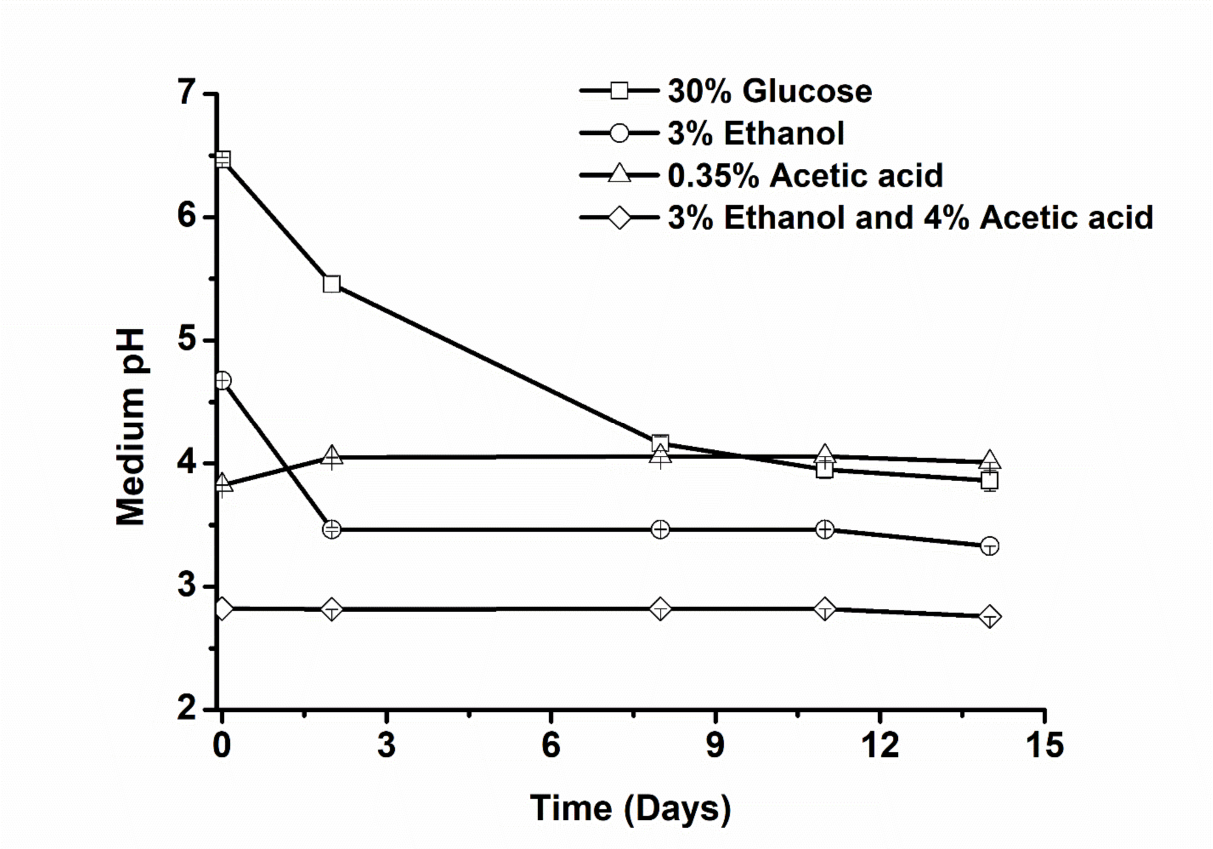

Figure S2: pH changes during cultivations in MA/9 containing 30% glucose and PY medium containing 3% ethanol, 0.35% acetic acid, 3% ethanol and 4% acetic acid. The cultivations were conducted at 30°C and 230 rpm for 14 days. Averaged values and standard deviations from duplicate cultivations are presented. In some cases, the error bars are smaller than the symbol

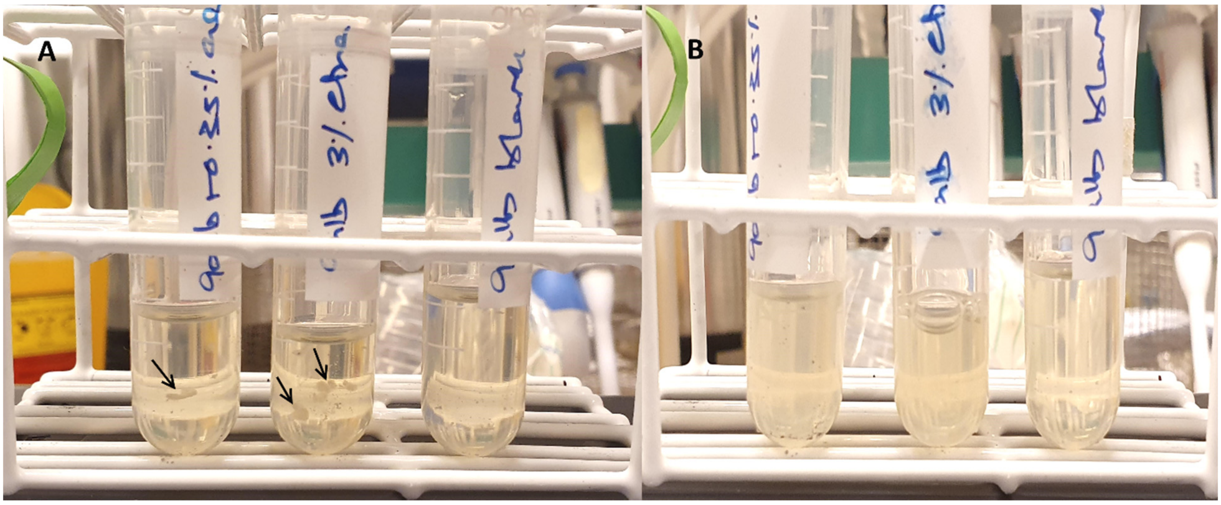

Figure S3. BC pellicle formation in PY medium containing 0.35% acetic acid, 3% ethanol and 4% acetic acid (A). Verification of BC pellicle, produced after 14-day cultivation in PY medium individually containing the above-mentioned carbons sources, were conducted using 1% cellulase. Lysis of BC pellicle and release of cells after the cellulase treatment is shown in B. The images were cropped and brightness and contrast were adjusted to improve clarity.

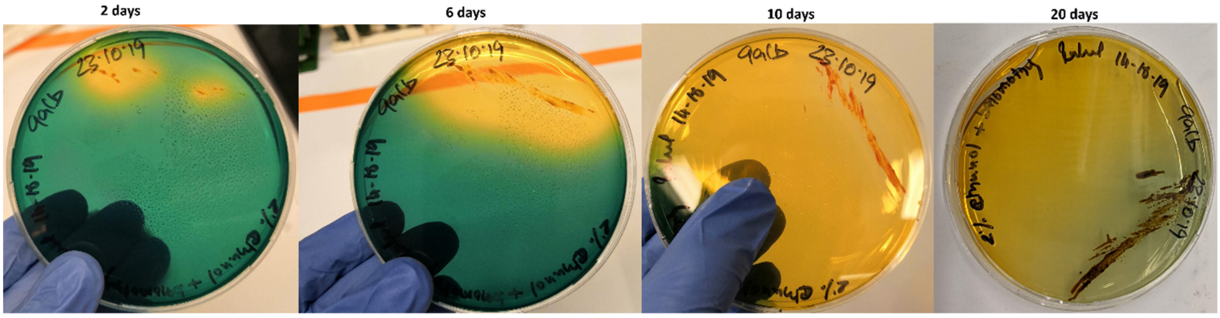

Figure S4: Overoxidation of acetic acid by ENS9B. Single colonies were streaked onto PY medium containing 2% ethanol and 0.0002% bromothymol blue as the pH indicator. Figures (from left to right) shows the acidification (blue to yellow coloration) and neutralization (yellow to blue) of PY agar during 2, 6, 10 and 20 days of incubation at 30°C. The images were cropped and brightness and contrast were adjusted to improve clarity.

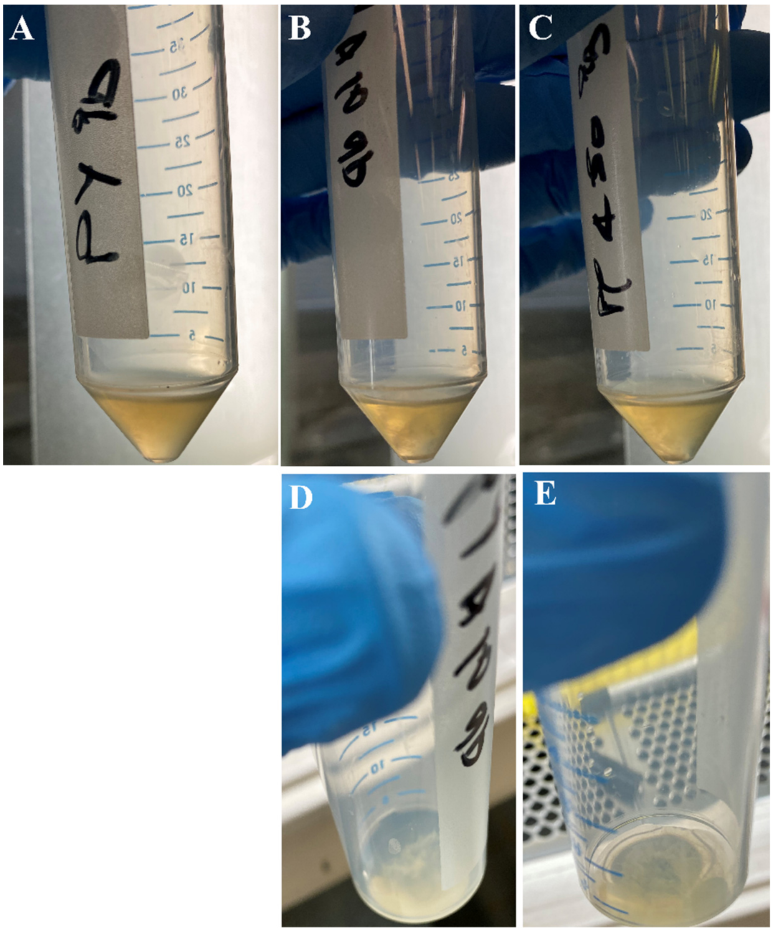

Figure S5: BC pellicle production by *K. rhaeticus* ENS9b cells statically grown in 50-ml tubes containing 10 ml (A) PY substrate control (i.e., devoid of acetate), (B) PY medium supplemented with 10-mM acetate and (C) PY medium supplemented with 50-mM acetate for 14 days at 30°C. (D) and (E) are the top-view images of sub-figures (B) and (C), respectively, showing the cellulose pellicles. The images were cropped and brightness and contrast were adjusted to improve clarity.

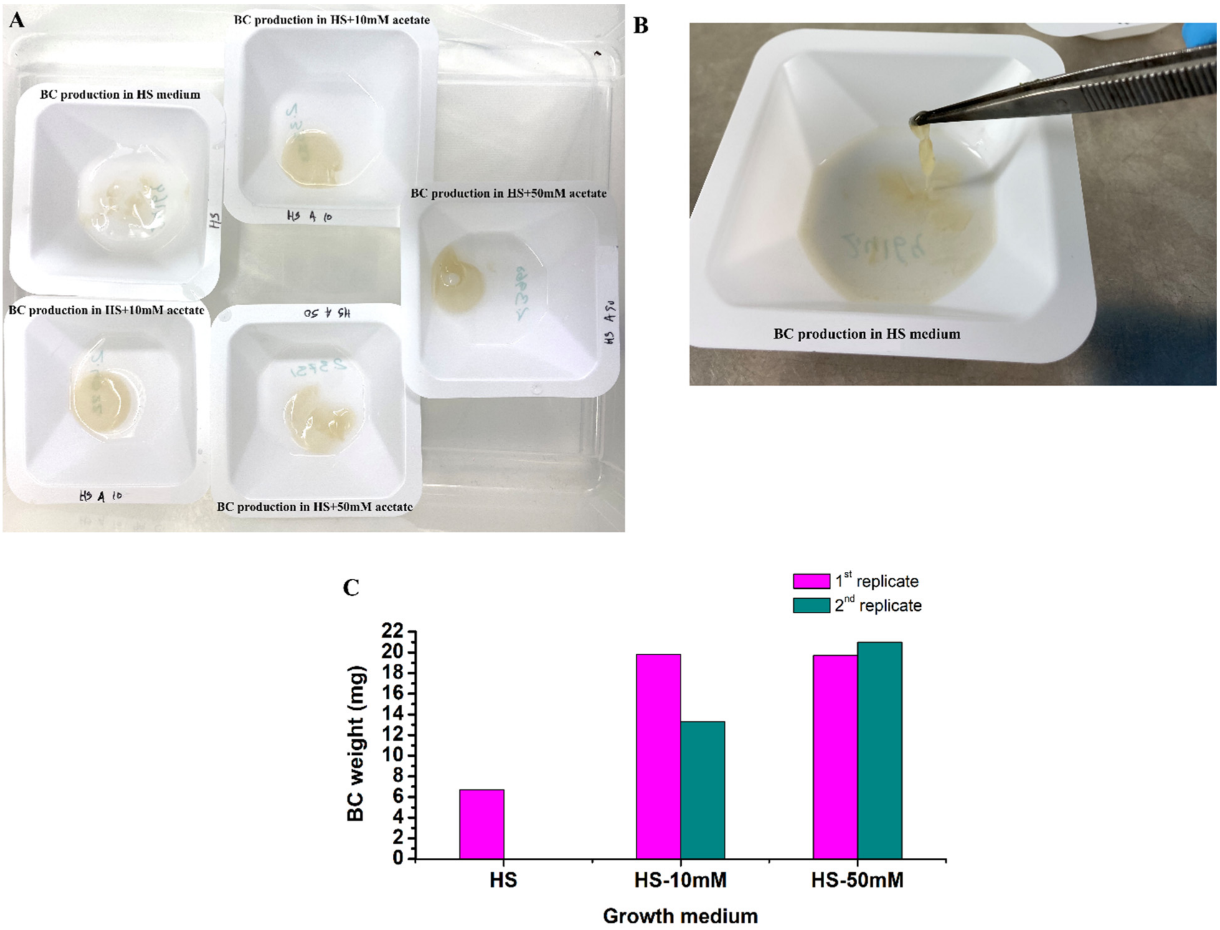

Figure S6. BC production by *K. rhaeticus* ENS9b cells grown statically in HS control (HS, without acetate) and sample (A10 and A50, medium containing 10-mM and 50-mM acetate) cultivation medium. (A) and (B) presents the pristine BC pellicles and the fragile pellicles produced from the control cultivation, respectively, placed on 46 x 46 x 8 mm pre-weighed weighing boats. The images were cropped and brightness and contrast were adjusted to improve clarity. (C) BC pellicle weights produced from HS medium with and without acetate supplementation. The BC pellicles produced from respective cultivations were collected and dried O/N at 60°C. The graph presents the BC weights (in milligrams) obtained from each replicate. The pellicles were not treated with alkali and MQ. Thus, the medium components present in the pellicles may alter the titers and thus, the BC weights are presented.

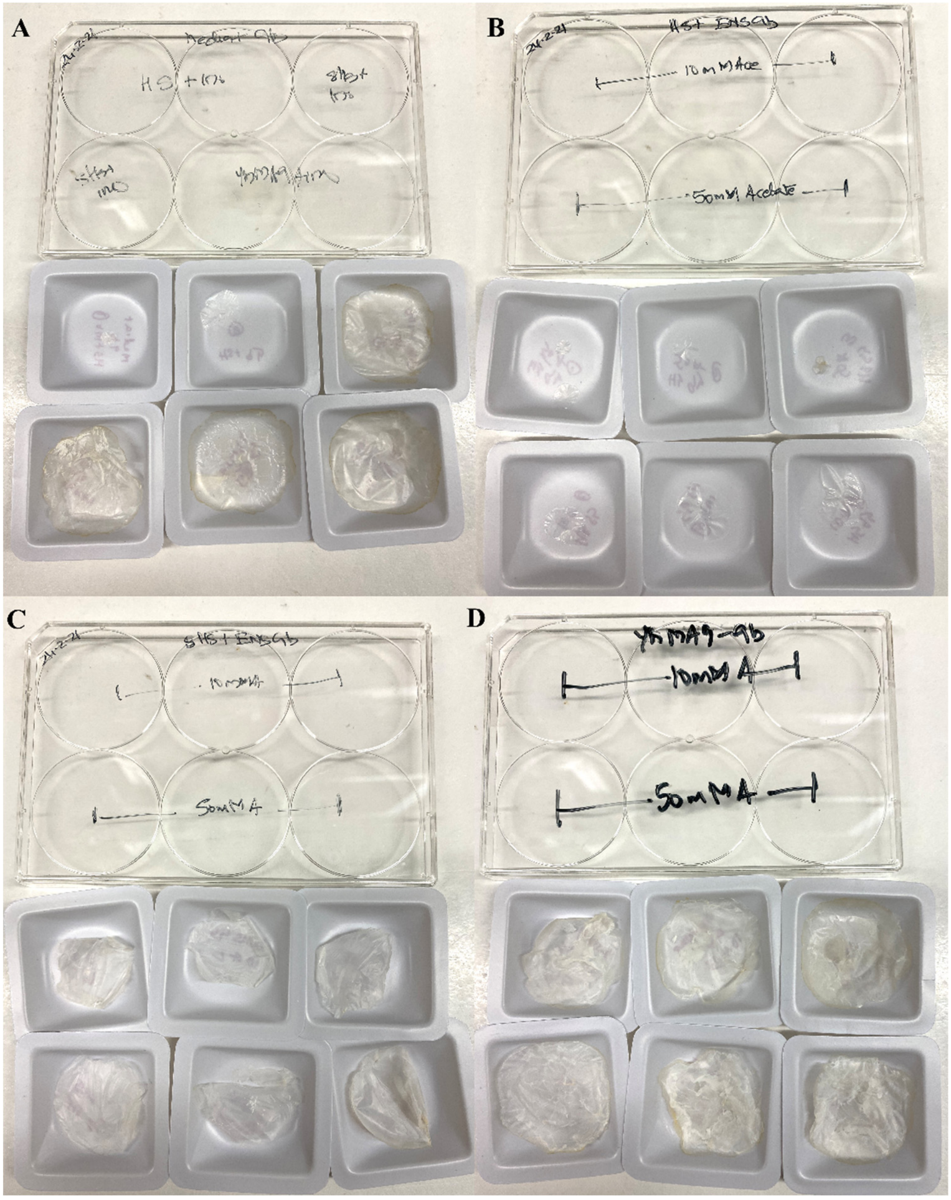

Figure S7. Dried BC sheets. BC pellicles produced by *K. rhaeticus* ENS9b grown statistically in (A) control cultivations devoid of acetate, (B) HS medium, (C) sHS medium and (D) YhMA/9 containing 10-mM and 50-mM acetate. The BC pellicles washed with alkali and ultra-pure water were placed on 46 x 46 x 8 mm pre-weighed weighing boats and dried O/N at 60°C. The images were cropped and brightness and contrast were adjusted to improve clarity.

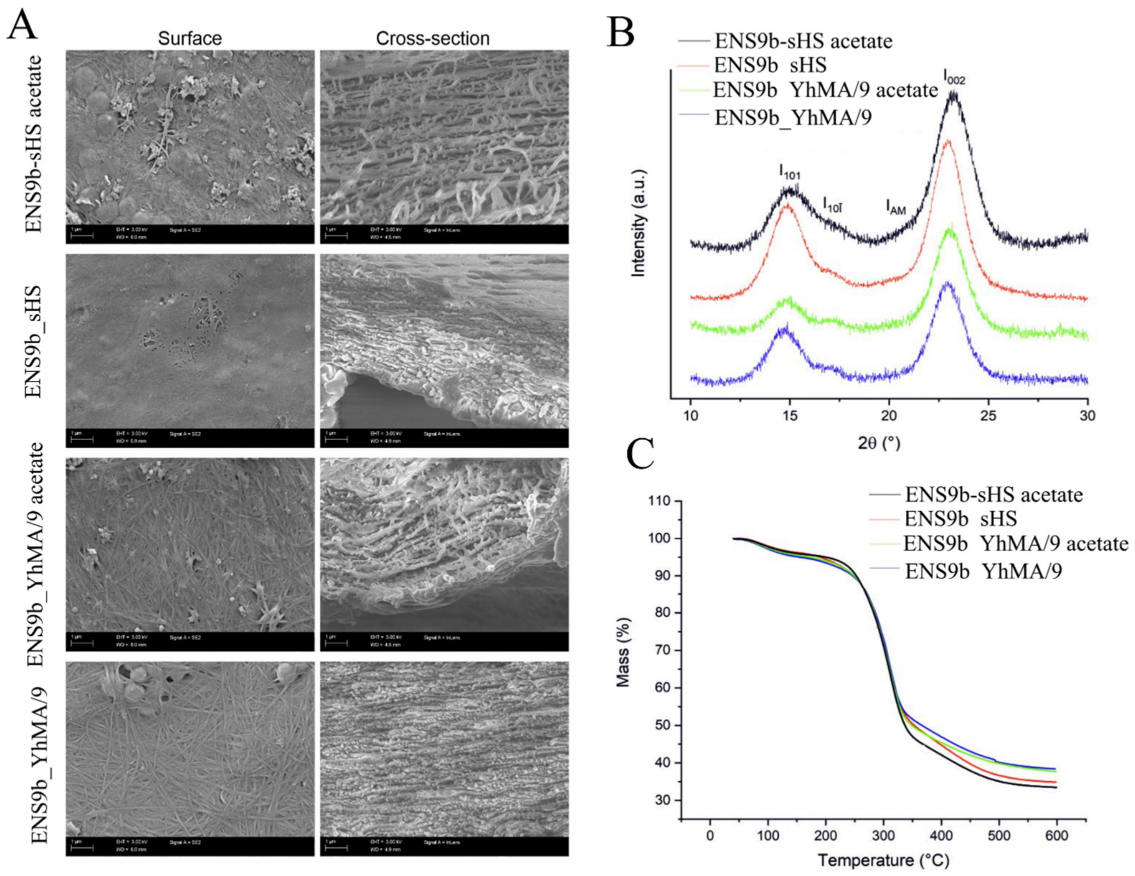

Figure S8. BC film characterization. (A) TGA (B) XRD diffractograms and (C) SEM analysis of BC films synthesized from acetate supplemented sHS, YhMA/9 medium and respective control cultivations devoid of acetate (ENS9b_YhMA/9 and ENS9b_sHS).

Supplementary Table

**Table S1** General genome and assembly statistics of *K. rhaeticus* ENS9b genome	
Attribute	Value
N50 (shortest sequence length at 50% of the genome)	200461 bp
L50 (smallest number of contigs comprising of 50% of the genome)	6
Contigs unplaced in the genome	7 (90 kbp)
Bowtie2 mapping	94%
Chromosome	3645912 bp (GC content, 63.1%)
Origin of replication	At genomic position 1099952:1100308 bp
Number of protein-coding genes	3302
Number of repeat DNA	120
Number of rRNA	3
Number of tRNA	46
Number of gene annotations to KEGG orthology	1646
Predicted plasmids	5 [p9b_1 (18826 bp, GC 59%), p9b_2 (13375 bp, GC 60%), p9b_3 (9758 bp, GC 60%), p9b_4 (3007 bp, GC 66%) and p9b_5 (2295 bp, GC 67%)]

Amino acid sequences

Enzymes involved in carbohydrate uptake and metabolism

>Carbohydrate-selective porin 1, OprB family (encoding gene at position 1261539:1263020 bp)

MCVVVSFYCNLSWARKPVPDHAARTQSGLLVSQPHAEVRETPAAPHASVSTRLNSIFRTEGLSILLTNR
EDALRVRDLSAGYYASSEAPGHILPQIGALRSRLQNRGVTFAFTYKGEAMADVGGGISRGMDYVHELT
LQTQFDLGRLFGFTGWTVHTLLMERVGREVSHDRVGDYNISLMEVYSLSGHSVAHLTDMYAQKSFLH
NTVDLAFGRMALTHVFATSPLLCSFMMTCSAPVAIKLDAGFSVYPKATWGGRVRLRPTRDTLVQIGAY
SVSPLNEDISGWAWAGEKSTGLMIPVEFAWQPFFGPRKLPGHYVLGFAHDTTRYADNIGDVPAGSAIR
PHGGEARDTFYLEADQMLYRKGGASQMAGGYVLAGYIHNTPDVSVISDEFYVGSSLLGIIPHRPHDRF
GVMYSYYRMSPRTELGQQLRMDAGMALGVHVNGPQTHAAVLEAYYGVPVYPGILVQPEFQYMMRP
GETAHIPNAEVVGLKVIGTL

>Carbohydrate-selective porin 2, OprB family (encoding gene at position 1287655:1289154 bp)

MCVIKTCANNENKSAIKWNFIPVRIRLLAVAVGMLFIGCTLTSEVLAQTETAKTAFTTHYDPAAFPMTE
MGQVAIGPLVPMLTASPHLFGDWGGIQPWLTNRGIFLNVTVNEEYMGNVTGGRQRANVAAGQVAG
ALDIDWQRLAGVPSFWTHMLVVNGHGSSLSNAIGDSITNPEEIYGARGNVVAHLVDMYADKGFLRDR
IILSVGVIPTGSFFNQDYLACSFMNVSVCGNPAPSKYVPGGRDWPSGNLGAVLRVRPTLRTYIMGGIFAV
SPHAYNGGISGWALGQDGLGKLSSQVEIGWSPSFGRHHLLGHYKIGYWYDNSRYPNLYADINGNSFQ
ATGLPRRYESGMNAAWFLFNQMIHRSGDGLANGLIVIGGADYTQGSQVAMRDHEWIGLLQSGTPWG
RPLDQVGAMFQYMEMGHTVTLQQESSLALGLPYLSNQWGAVYGIQSHENVWEAFYSIHVARGTAFQ
PDFQYLQRPGATTTFHDAAVIGFQFTTNL

>Carbohydrate-selective porin 3, OprB family (encoding gene at position 1433875:1435551 bp)

MTHSILTKRILAGPSRFVPPFHTPHGSMCRQGLCAASCALGMGLIFSTSGMAQTQIEEDRARLGNSTPLI
QQIDPANVRDSERAQADVEEKAAGDGDKLDTSGNLLGNMWGARPWLYKHGITFDIQEVDEVWGNG
TGGSASGNDGAGGSGTGPAYDGVTMPTLTIDTEKLFGLKGGLFNVSALQTRGRSISQDHLANFNPVSG
FEADRSTRLFELWYQQSFLKGTLDVKIGQQDLDTEFLISDYASLYLNANFGWPMAPSVNLYGGGPSWP
LASPAIRIRYRPSEKFTFMFAAADDNPPGNRYNSTPIQQATGSVSGYNSDPTSQTYDGANGTNFNMGTG
ALLITELQYALNPQPADMSNVTKNPGLPGVYKLGGYYDTAKFADYRYNRNGGSLGAASVNPDSTESD
LTPRWDRGNWMVYGIIDQMIWRPSIRSARSVGIFARATGNSGDRNMISFAVDAGINLKAPFRGRDNDT
LGLGWGIGRASSGQRQFDRDSGSYVQGNENHLELTYQAQVTPWMVMQPDFQYIWHPSGGVADWTG
LRRVGNEAVFGLHSSITF

>Carbohydrate-selective porin 4, OprB family (encoding gene at position 2853448:2854926 bp)

MKYAIDTRMTRPFPRLFVSLPLFFLALFALCESASAQAIPGEGQGVVGAQPSLNVNSRRNVSVNDPGSFF
SAPYGNQHFFGDWAGLQPFLLKHGVHIEADVHEELAGNFRGGAKQGVTDAGQVGVEIDIDWHKLA
GAPKNFWTHTMIVNGHGRNASTDYIHDSLAGVQQIYGARGNVVAHLVYMYAEQSLFHNRLDISAGW
IPVGSFFAASPLFCDFMNVSICGNPAPGKYVPGGRDWPSGNLGVVMRVMPTVDTYIMASMFAVSPHSY
NGGISGWSWAQSGLGKFSTPVEIGWLPEFGRNHLAGHYKAGYGYDNSQYNDLYSDINGNSAVLTGQP
FRKQSGVSTAWFQADQMIMRNGAGPTNGLILLAGYMYTSGKVSAMKDHTWAGIVETGAKWGRPLD

TVGAMFHYYEMSRASILQQESSVLAGTPFLNNQWGKVWGIQTHENVYEVFYNIHTARGMSFQPDFQYINRPGGTTTYHDAAVMGLQFNCIL

>Carbohydrate-selective porin 5, OprB family (encoding gene at position 3040876:3042462 bp)

MKIPDPPPPRWTALSRLLFLSCMLIGPCGATTAHAQVPRTQIADDRARLGNSTPLIQQIDPATVRDSERAQADAQAPPSADALDTSGSLLGNMGGARPWLYRHGISFDLQDVNELWGNATGGSASASDGAGGAGTGPAYDGVTMPTLTIDTEKLFGLKGGLFNVSALQTRGRSISQDHLADFNPVSGFEADRSTRLFELWYQHSFLGGKLDVKIGQQDLDTEFLISDYGALYLNANFGWPMGPSLNLYAGGPSWPLASPAVRVRYRPDETFTFMFAAADDNPPGNSSNSFVIQNGGNRADPTSQTTNDGSGTQFNMGTGALLITELQYALNPQPADMGSATRNPGLPGVYKLGGYYDTARFPDYRYNMQGRPLGTYGGMPRQDRGNWLLYGIIDQMIWRPSLASPRSLGVFARATGNSGDRNMISFAIDAGLNLKAPFRGRNNDTIGLGWGIGRASSGLREYDRTSGALLQGSENHLELTYQAQVTPWMVMQPDFQYVWHPSGRVADWSGLRRVGNEVVCGLHASLTF

>Carbohydrate-selective porin 6, OprB family (encoding gene at position 3439017:3440453 bp)

MSVPGHAQTIPVAARDVWQADEIPASESQDTPSAAGGSLLGDMGGLRPWLARYGMTLGIQDVNELWGNATGGIASTSGDGRGAGTGASYIGVTMPDLTADLEKLMGLVGGTFNVSALQIRGRAITQDHLADFNPISGFEADRSTRLFELWYQQSFLKDTLDIKIGQQDLDTEFLISDYASQYLNASFGWPMGPSANLYAGGPSWPLAAPAIRLRYRPGGNYTVMFAASDDNPSGNRDSNVFGVTSNPADPTSQTMNDGSGTQFNMGTGALLITEIQYALNPRSGDRPGAVRHAGLPGVYKLGGYYDTARFPDYRYNMQGQSLGAYGGTPRWDRGNWLIYGIVDQLIWRPSPEGARALGVFVRATGNSGDRNIISFAIDAGLNLKAPFAGRTNDTLGLGWGIGRATSGVRAYDRARGALVQGNENHFELTYQAQVTPWMVLQPDFQYVLDPSGGLGNPSRPMRRIGNEAVFGLHTNVNF

>Carbohydrate-selective porin 7, OprB family (encoding gene at position 3440518:3441825 bp)

MVVFLAVLSPAYAQTVPVSVPSAGVTPRQSVPDHIDPRTRLSGDWGGMRNWLLAQGIDIRISDTDELWANPVGGAQASENYIGSTAVEMVTDLHALTGLALGTFDISAMEIRGRPFSNAPLYVFNQTSNIEADDNGRLYELWYSQKFLRERLALRIGKLDLGHDFMVSSVGLTFLNASFSWPIMPDNDLYDQGPVSPVTTPAIRLRYTLSPRWNFLFAAADDNPVGGPFINVRDPWNQNRDPGGTRFSFSTGALFFGEVQYRRDISGRQGTYKLGGYFDTGRFPDQSDFAKSHKTNWAVYGIVDQTLRHFGRITTLDAFIRGNWTADTDRNQIVYAVDGGIALSAPFHRRGDVLGLGAGLGAASPCLAAADRRSGLPAQGNEYHLELTYQAQVTPWFMLQPDIQGIVSPSGGVLDDRGRRVHDEAIFGLHGSVTF

>Aquaporin Z_1 (encoding gene at position 1386137:1387120 bp)

MKYIEIHGKRRSAYPTCRSTLFPHPPCSFKNIRGLEKGQKAHSPFPSNDNKRIKWANMLKNRQFLGELIAECIAVAIIVMIGDSVAAMYALYDPSPYKLAYWGVCIVWGLSVTIAIYITGSVSGTHANPAVSVALALYRGFPWRKVPAYCAAQILGGVIGAALVYTLYQPVIDHYNQLHALTRADGGAAGVFFTHPGEFITPFHAFVDETILTGLLVFGIFAVTCEYNTVAPQANSGALIIGLLVASIGACSGYLEAWAINPARDFGPRLFCFLAGWGDSALPGPDHFWWVPVAGPVLGGVLGAGCYQFLIRPFIPRQASPAVPPVS

>Aquaporin Z_2 (encoding gene at position 1847677:1848606 bp)

MRLPHLHLPYLHVHVHDRPLAGDPHPHNPFHWKLYFCETVATAILMVLGLSGVILLTAPGSPLSPPLLHHPYVQTALCGLVFGLSGTAAAMTPFGKVSGAHINPSVTLAFSLAKRIGGVDALNYMIAQVIGAFLGTAVVYGLGRLVTWWGSMAVAVRYGATVPYGRISIWWAMWSEMFVTAALIAMLYWLAAHPRWKFITP

WSGGLFFLIMNPFTAWLSGNSVNFARTLAPALFSGQWTGLWVYVVGPFAGASLAVMAIRMNLMGRL
HLLEARLVNFGHHGRVPGLDDPHRKLNHPDDPDHVPPGTGPA

>Aquaporin Z_3 (encoding gene at position 3161790:3162686 bp)

MTATRPPGGSAITDRPLAGAPHPDHPLHWKLYGCEMVATMVLMLCGIVSVTLLTTPQTPVGRMLAPH
PVIQTMLCGLFFGLAGTVAAYTPFGRVSGAHVSPSVSLAFSLARRLGWVDLCGYVAAQMLGACLAAV
LLAGVGHVLPVWGKMAQDATFAATVPFSGVAVVWPLVTEMVLTCLLVIMLCALAAHPVLKWFTPW
AGGVFFCLCNPVSAWLSGNSSNLARSFGPAVVTGQWDSFWIYALGPFTGAAMAIWLLRSRLLGRIEVE
EARLVNFGHHGRIPSLLHPGRRMEGGPAKYD

>Quinoprotein glucose dehydrogenase_1 (encoding gene at position 1924182:1926572 bp)

MNTPLRSAPLLAAAIGVCALAGLYLLGGGLWLCAIGGSFYYVVAGALLLVTAFLLFRRQAVALTVYAA
LLLGTMAWAVNEAGLDFWALAPRGDILVPIGIVLLLPWVTRHLQPATPGARLPLLGAIGAAVVVIGAA
LMGDPQDIAGSLPPVAQNAPEPGDAHQMPDEDWQAYGRTQFGDRFSPLRQINTGNVSRLKVAWTFR
TGDMRGPNDPGEITDEVTPIKVRDTLYLCTPHQILFALDARTGKQRWKFDPQLQYNPTFQHLTCRGVS
YHEDRVEAQADGAPAPAPAECARRIFLPTNDGQLFALDAESGARCASFGNNGVVDLRDGMPVRTLGF
YEPTSPPVVTDTTVIVSGAVTDNYSTHEPSGVTRGFDVHTGALRWAFDPGNPDPNEMPSEHHTFVPNS
PNSWITSSYDAKLDLIYIPMGVQTPDIWGGNRGADAERYASAIVALNATTGRLVWSYQTVHHDLWDM
DIPAQPSLVDIRNEHGEVIPTLYAPAKTGNIFVLDRRNGQLVVPAPEQPVPQGAAPGDHLSPTQPFSRLT
FRPSQKLTDADMWGGTMFDQLVCRIMFHRLRYEGTFTPPSLQGTLVFPGNLGMFEWGGLAVDPERQI
AIANPIAIPFVSKLIPRGPNNPATPDRSLPSGSESGVQPQFGVPYGVDLHPFLSPLGLPCKQPAWGYMSGI
DLRTNKIVWKHRNGTIRDSAPLPLPIWMGVPSLGGPLTTAGGVAFLTSTLDYYIRAYDVTTGRVLWQD
RLPAGGQSTPMTYAVDGRQYIVTADGGHGSFGTKLGDYIVAYTLADQN

>Quinoprotein glucose dehydrogenase_2 (encoding gene at position 2594170:2596791 bp)

MRQDLPFESHAREIPRNPSGAACLHHAVQTRWPHTGIESIMFPRPIENFRKNAGMREGKQKLFSIITSAL
LALVGLFLFFGGAQLMVLGGSWFYVLAGCLLLAVAVTGVKAPQLAMRVYAAFLVVATIWALTETGFN
IWGLEVRLLAPIGFGAWMLLPWVWRSGGNWLADKRDMLGAIGLAVVVLVISCFTSYSIDGTVPDDRM
AAQGQADPAADGVADADWQAYGRTMGGDRYSPLAQITRGNVSHLKRAWVTRTGDVEQDGEGTVA
GPDQGHEFNLEVTPLKVGDTLYMCTPHSWVMALDATTGKVKWKFDPHPDTADLAKNVYLACRGVS
YYRIPDEIQTSCRTRIYSPVADVRIVALDAETGKPCDDFGDHGFISMRAYLGHVPHGFHFITSPPLVAKN
RLITGGWIFDNQANFEPSGAIRAFDATTGEIAWAWDAGHSPETWKPGPTDVLTRDTPNAWGVYTADP
ALGMVYIPTGNAPPDNWGGSRRPFDDATSSATIALDIVTGERRWTYQTVHHDLWDMDIPSGPSMVEL
PGPNGESIPALVQSTKRGEFFVLDRRTGAPVPGYPVEERPVPTAGAAPDDRVSPTQPYPAAMPSLTPPN
LKESDMWGATLLDQMICRIQYRQSAYEGQFTPPHVGRTTIVYPAFYGVVDWQGITIDPQRKIMLANAS
YLPFRIKLEKRQGLEGKGVLPRWNGQGEEPAAKGDALSVSPDYGTPYIALTNPWLNPLQIPCKGPVWG
TITAIDLVTKKIVWQHPVGTTRDTGPFRTHNNLPLPTGMYNIGGSIVTRGGLVFMGATADDYLRAFDL
ATGKVIWTDRLPAGGQATPMSYEIGGKQYVLIAAGGHGGLGTRSGDYIIAYTLDGEKGAASAQ

>Glycerol uptake facilitator protein (encoding gene at position 1285133:1285972 bp)

MNRVFLGELISEALAVFIIIAFGDSVAAMYLLYDPSPYLHAYWGVCIVWGLGVTVAIYATGSVSGTHAN
PAVTLALATYRAFPWKKVIPYWFAQIVGGFLGAALVYALYYPVIDHFNDMHQLARMSGGGAGVFFTA

PAPGITLWHAFSDEIILTAILVFGIFAITEQYNEMAPGANFGALIIGFLVAAIGASMGYLEAWAINPARDL
GPRLFAYVAGWHEAALPAAGHYWLVPVAGPLIGGLLGATAYEYLIYPFLPAQLRARQALQGKIAEETII
IGE

>Glycerol kinase_1 (encoding gene at position 1286008:1287507 bp)

MTDYVGAIDQGTTSSRFMIFDRKGTIVSVAQKEHRQIYPRPGWVEHDPIEILENTNEMIGAAMARANL
GAASLVSVGITNQRETAVLWDRTTGLPLHNAIVWQDTRVDQLVSEYGRDGGQDRFRAITGLPLASYFA
GLKLRWLLDNVEGARQKAESGQALFGTVDSWLSWNLTGGTRGGIHITDVTNASRTQLMNLKTCAWD
EDMLAAFSIPAACLPRIVPSSQVYGTITIPSLQGTKLAGILGDQQAALVGQTCFSPGEAKNTYGTGSFLL
MNTGTDPVQSKAGLLTTVAYQFGTEEPRYALEGSIAITGALVQWLRDNLKLFDLATQIEPLARSVADN
GGVYIVPAFSGLYAPYWKENARGVIVGLTRYVTRAHFARATLEATAYQVRDVVAAMEEDSGIVLRSLK
TDGGMVANELLMQFQADILNVPVVRPKVVETTALGAAYAAGLAVGYWKNIDDLRANWEVDRTWQ
PTMPPEQRARYLAWWKKAVQKSFDWAE

>Glycerol kinase_2 (encoding gene at position 1389048:13890547 bp)

MNKKNRILAIDQGTTSTRSIVFDRDIAALSVARVEFAQHYPNQGWVEHDAEEIWSNVLSTAREAIAKV
GGPATIAGIGITNQRETIVVWDRKTGSPVHRAIVWQDRRTTPVCMRMHEQGYEPLVRERTGLLLDPYFS
ATKIAWILDNVEGARARAERGELACGTIDSFLLWRLTGGRVHATDTTNASRTLLFNIHTCAWDDELLA
LFNVPRAILPEVRTNSEIFGETEADLFGEPLKVAGMAGDQNAAMVGQACFKPGTAKATYGTGCFALL
NTGTTPVNSENRMLTTIAYRIGDETVYALEGSIFVAGAAIRWLRDGLGLITHASQTDDMATRVPHSHGV
YMVPGFVGLGAPHWDPDARGLICGMTLGSTAAHIARAALESVAYQTLDLMEAMREDGGGKLNALRV
DGGMSVNDWFCQFLADMLQTPVERPRQVETTALGAAFLAGLATGVWSSIPELEGTWTRGHLFRPTMD
KAQRDSMVAGWHLAVRRTLSSTVAA

>Glycerol dehydrogenase_1 (encoding gene at position 1201435:1203437 bp)

MRRYRFLAMGMAAAWLCLPVSSHAQFAAAGSGGPPGASIPGPGRADEPAEQTPADPGYFAGPSPYAP
QAPGVNAANLPDIGSVAADSLPAMLPQAGAAPAPGDWATYGRDDAQTRYSPLAQITPENVSHLQVA
FTYHTGSYPRPGQTNKWAAETTPIKVGDGLYMCSAQNDIMKLDPATGKEIWRRNLGEKYEAIPYTAAC
KGVTYFTSSQVPQGQPCHNRILEGTLDMRLVAVDAATGEFCPGFGHGGQVNLMQGMGESVPGFVAM
TVPPTVVNGVVVVNHQVLDGQRRWAPSGVVRGYDAESGRFVWAWDVKHPDDHDQPTGNRHYSRG
TPNSWAAMTGDDALGLVYVPTGNAAGDYFSGYRSAAENAVSTSIVALDVRTGSPRWVFQTVHKDVW
DYDMGSQVTMMDMPGTDGQTVPAIIVPTKRGQTFVLDRRTGQSILPVEERPAPAGDVAGDTRAPTQP
WSVGMPSLHMPDLREQDMWGMSPIDQLYCRIKFRRAHYVGEFTPPSLDRPWIMYPGNNGGSDWGSM
AYDPQTGILMMLREYGGKPLVEWLVLLLGVVIFLVGLVFVAGGAELALLGGSLYYLLCGLVLLASGTA
MLMGRTLGALLYLAALAYTWAWSVWEVGFSPVDLLPRAFGPTLLGLPVLFSIPVLRRMATRRAIGGIC

> Glycerol dehydrogenase_2 (encoding gene at position 1673449:1676036 bp)

MSRTPMPRGLLLGATIITGLSCATALAQVPPADQPPGTGNATVPPAQPTPPADAFSAPSPNGPQAPGV
NAANIPDGPPAEPSEATPMVEQVVANPAHDDWPGYARDDRATRYSPLDQITPANISHLKRAFIYHTGS
KPGPGQVNKWAAETTPIKVGDGMYMCSALNDMMRIDPATGKEVWHFHANEKYESIPYTAACKAVV
YYTSSTVPEGEHCHHRILEATLDERLIEVDSEDGKVCEGFGWHGMVNLMKGMGESVPGFVAETAPPPI
VNGAIITNQEILDGQRRWAPSGVIRGYDAETGRFLWAWDVNRPHEHGEPKEGEHYSRGTPNSWAAMI

GDNALGLVYVPTGNSAADYYSAMRSPEENKVSSAVVAIDVKTGEPRWVFQTVHKDVWDYDIGSQPTL
MDFTKPDGSSVPALIMPTKRGQTFVLDRRTGEPVLPVEERPAPSPGMVPGDPRAPTQPWSVGMPRLGF
APLKEADMWGMSPIDQLYCRLKYRRAHYVGEFTPPSIDKPWLEYPGYNGGSDWGSVAYDEKTGILIAN
WNNTPMYDQLVSRKKADQLGLMPVDDPNYKPGGGGAEGAGAMAETPYGIVVSPFWDEYTGMMCN
RPPYGMITAIDMHTQKVLWQHPLGSARANGPFGLPTHLPLTIGTPNNGGPVITAGGLIFVAATTDNMI
HAFDIHTGREVWNDVLPGGGQATPMTYEYKGKQYVAIMAGGHHFMMTPVTDDLVVYALDNVNMS
AFVTPQTPAGWATLGLAVIIILLGLPLLYMGAELAFIGGSWYYVIVGLAVTVAGLLMAMGRVAGGLLY
LAAVAFTWLWALWEVGLDGWGLLPRVFGPTLLALGVLACLPVLKRAEAARSTLVREVA

>Dihydroxyacetone kinase (encoding gene at position 1848749:1850380 bp)

MKRFFNTRETIVTEALDGFLRSAAGQHLCRLDGYPDTCVIMQREPERGLVSVISGGGSGHEPAHAGFVG
RGMLTAAVCGALFASPCVDAIVAAILATTGEAGCLLVVKNYTGDRLNFGLAAERARALGKQVEMVIV
GDDIALPDSATPRGVAGTVLAHKLAGYGAAQGWPLTRVAQFVRDGAGRMRTIGMALEDCNPYEPGR
ASRLAADEAELGLGIHGEPGAQRIALASASDLMRRVTETLEASLPATVRNTRFAVVLNNLGCVPEVEM
ALLLEAFSHTRLARRVSHVIGPAPLMTALDMNGFSLTLIELDESITHALQAPAQPPAWPGMVPLGSPAV
APMPVMPDAFPYPASTDPTVRAMLEHGAKVLIANEAPLNELDGKIGDGDAGSTFAGAAREITAALDR
LPMADPHQLMTTVSNILTQHAGGSSGVLFAIMFSAAGRSRAPWREALREGLEHMMACGGAKPGDRT
MIDALYPALEALARTGSLTDAALAARKGADATTTMETARAGRAAYVPSAHVRNVPDPGAEAVARLL
EGLARG

>Triosephosphate isomerase (encoding gene at position 2116868:2117611 bp)

MKQIIVGNWKMNGLSADADALVDALATGLAALPARADVVVCPPFTQLARLAPKLKAAGMALGAQD
CHKDRSGAHTGDISAPMLVDLGVEYVILGHSERRAEHGELDETVREKAVAAMAAGLTPIVCVGENAD
QRDSGDSQEKVGWQIQGSLPQGFTGIVAYEPVWAIGTGQAASQQDIADMSAFIRAELVRQFGEAGKTI
RILYGGSVNERNVTDILPVENVDGALVGNASLKADAFVPLVRAARAS

Enzymes involved in BC biogenesis

>bcsA1 (encoding gene at position 666919:669156 bp)

MSEVQSSAPAESWFDRLSNKILSLRGASYIVGAIGLCALLAATTVTLSVNEQLIVALVCVVVFFIVGRRKS
RRTQIFLEVLSALVSLRYLTWRLTETLDFDTWLQGTLGVTLLLAELYALYMLFLSYFQTISPLHRAPLPLSP
NPEDWPTVDIFIPTYDESLGIVRLTVLGALGIDWPPDKVNVYILDDGKREEFARFAEECGARYIARPDNA
HAKAGNLNYAIQHTSGEYILILDCDHIPTRAFLQISMGWMVEDKKIALMQTPHHFYSPDPFQRNLAVG
YRTPPEGNLFYGVIQDGNDFWDATFFCGSCAILRRKAIEEINGFATETVTEDAHTALRMQRRGWSTAYL
RIPLASGLATERLVTHIGQRMRWARGMFQIFRVDNPMLGPGLKLGQRLCYLSAMTSFFFAIPRVIFLASP
LAFLFFSQNIIAASPLAVLAYAIPHMFHSVATAAKVNKGWRYSFWSEVYETVMALFLVRVTIVTMMFPS
KGKFNVTEKGGVLENEEFDLGATYPNIIFAVIMAIGLMRGLFALAFQHLDIISERAYALNCVWSVISLIIL
LAAIAVGRETKQIRHSHRVDARIPVTVYDYEGNSSHGITQDVSMGGVAIHMPWRNVTPDQPVQTVVH
AVLDGEVVNLPATMIRCANGKAVFTWNITSLPIEASVVRFVFGRADVWLQWNDYEHDRPLRSLWSLIL
SIKALFRKKGRMMIHSRPQNKPIALPVERREPTSSQGGQKQEGKISRAAS

>bcsB1 (encoding gene at position 669157:671572 bp)

MRPRDMKMVSLIALLVFATGAQAAPVASKAPAPQPAGDNLPPLPAAAPAAAAAPAGQQPAGAASA
APAVDPAAASAADAMVDNAENATGVGSDVATVHTYSLRELGAENALTMRGAAPLQGLQFGIPGDQ
LVTSARLVVSGAMSPNLQPDNSAVTITLNEQYIGTLRPDPSHPAFGPLSFDINPIFFVSGNRLNFNFSAGS
KGCTDPSNGLQWASVSEHSELQITTIPLPPRRQLSRLPQPFFDKNVRQKTVIPFVLAQTFDAEVLKASGIL
ASWFGQQTDFRGVNFPVFSTIPQTGNAVVVGVADELPSALGRPAISGPTLMEVANPSDPNGTILLVTGR
DRDEVITASKGIGFSSSTLPVAARMDVAPIDVAPRAPNDAPSFIPTSRPVRLGELVPVSALQGEGYTPGVL
SVAFRTAPDLYTWRDRPYKLNVRFRAPDGPIVDLARSHLDVGINNTYLQSYSLHEKDSVVDQLVQRFG
GRGQTSGVQQHTLTIPPWMVFGQDQLQFYFDAAPLTQPGCRPGPSLIHMSVDPDSTIDLSNAYHITRM
PNLAYMASAGYPFTTYADLSHSAVVLPDHPNGTVVSAYLDLMGFMGATTWYPVSGLDIVSADHVND
VADRNLIVLSTLANSGEVSSLLSNSSYQIADGRLHMGMRSTLSGVWNIFQDPMAAINNTHPTEVETTLS
GGVGAMVEAESPLASGRTVLALLSADGQGLDNLVQILGQRKNQAKIQGDLVLAHGDDLTSYRSSPLYT
VGTLPMWFMPDWYMHNHPVRVIVVGLFGCLLVVAVLVRALLRHALFRRRQLQEERQKS

>bcsC1 (encoding gene at position 671575:675549 bp)

MNRRYVFSLSAGLLASSCMTVLVAVPLARAQQASTAMTGTQASGGSAAPRQILLQQARFWLQQQQY
DNARQALQNAQRVAPDAPDVLEVQGEYQTAIGNREAAADTLRHLQQVAPGSTAANSLSDLLHERSIS
TSDLSQVRSLAASGHNAQAVQGYQKLFNGGKPPHSLAVEYYQTMAGVPAEWDQARAGLAGIVASNP
QDYHAQLAFAQALTYNTATRMEGLARLKDLQGFRSQAPVEAAAASQSYRQTLSWLPVTPTTQPLMQ
QWLDSHPNDTELREHMVHPPGGPPDKAGLARQAGYQQLNAGRIAAAEQSFQSALQINSHDADSLGG
MGLVSMRQGDAAEARRYFQEAMAADPKTADRWRPALAGMEISGDYAAVRQLIAAHQYDAAKQRLS
ALARQSGQFTGATLMLADLQRTTGQMGAAEQEYQSVLARDPNSQLALMGLARVEMAQGKTAEARQ
LLSRVGSQYATQVGEIEVTGLMAAASQTSDSARKVSILREAMAQAPRDPWVRINLANALQQQGDMAE
ANRVMQPILSNPVTAQDRQAGILFTYGSGNDAMTRRLLAGLSPEDYSPAIHAIATEMEIKQDLASRLSM
VANPVPLIREALSPPDPTGARGVAVADLFRQRGDMIHARMALRIASTRTLDLSADQRLAYATEYMKIS
NPVAAARLLAPLGDGSGTGAGNALLPEQVQTLQQLRMGIAVAQSDLLNQRGDQAQAYDHLAPALQ
ADPEATSPKLALARLYNGQGKSGKALEIDLAVLRHNPQDLDARQAAVQAAVNSGRKSLATRLAMDG
VQESPMDARAWLAMAVADQADGHGHRTISDLRRAYDLRLQQVEGTRAVASGTGEQESLEPPSSNPFR
HHGYGRQTELGAPVTGGSYSMEATSPEASDQMLSSIAGQITTLRENLAPSIDGGLGFRSRSGEHGMGRL
TEANIPIVGRLPLQAGESSLTFSITPTMIWSGQLNTGSVYDVPRFGTDMATQAYNQYVSYISQNNSSSTL
HSELVKGGEAEAGFAPDVQFGNSWVRADLGASPIGFPITNVLGGVEFSPRVGPVTFRVSAERRSITNSVL
SYGGMRDPNYNTTLGRYARQLYGKELSSQWSEEWGGVVTNHFHGQVEATLGNTIVYGGGGYAIQTG
KHVQRNDEREAGIGVNTLVWHNANMLVRIGVSLTYFGYANNQDFYTYGQGGYFSPQSYYAATVPIRY
AGQHKRLDWDVTGSVGYQVFHEHSSPFFPTSSLLQSGAQYIADSYVQNATSSDYLSQETVNSAYYPGDS
IASLTGGFNARVGYRFTHNLRLDLSGRWQKAGNWTESGAMISAHYLIMDQ

>bcsD (encoding gene at position 675549:676019 bp)

MTTFNAKPDFSLFLQALSWEIDDQAGIEVRNDLLREVGHGMAGRLQPPLCNTIHQLQIELNSLLAMIN
WGYVQLELLPEDHAMRIVHEDLPQVGSAGEPAGTWLAPVLEGLYGRWITSQPGAFGDYVVTRDVDAE
DLNSVPSQTIILYMRTRSSSN

> bcsZ (encoding gene at position 664722:665759 bp)

MGRRSFLSVMAAAGSIPFLSTALAADDPAINAQWAIFRAKYFHPDGRIIDTGNSGESHSEGQGYGMLFA
ATAGDQATFEAMWSWTRANLQHKTDALFSWRYLDGHNPPVADKNNATDGDLLIALGLVRAGRLW
KRADYIQDAIAIYGDVLKLMTLQVGPYLVLLPGGVGFATKDSVTLNLSYYVMPSLMQAFALTGDARW
QKVMGDGLIIINQGRFGEWKLPPDWLSINRQNGHFSIANGWPPRFSYDAIRVPLYLYWAHMLSPDLLA
DFTRFWNHFGASALPGWIDLTNGARSPYNAPPGYLAVATCSGLSSAGGLPTLDKAPDYYSAALTLLVYI
ARGEGGM

> ccpAx (encoding gene at position 665756:666736 bp)

MTTPNKESTEATPRPVVDMDNPQDVTRMLSSGYGLSGEGFHYHSFTRPVMLDMTPELPHDSVDDTEH
HLDDNVTEHESAPATAPVFVFDAAPEPAAPPVVAEAAPPPPPPPPPEPAPPEPPPAPPPYTPVVTHVPPP
PPVEETPVPEPVAEAAAPARPRPVPPAQPAPDMASTGGRERRGLQPFAAPRAPSEPPVSSRAAAPATPF
VQADDWAPVPKAQQRRGQRPTGPGFFFAKGNDRVATARLFQPVAVARPASKPDSKVTTMTKFDKTA
QNAPTGRRPAPSDNSPTLTEVFMTLGGRATDRLVPKPSLRDALLRKREEETGQS

> bglX (encoding gene at position 676251:672452 bp)

MKPSRKSFLLSAVAWGLVAALPAHARHAATAGDPADDQARQVLAHMSLQDKMALLFSVDGGGFNG
SVAPPGGLGSAAYLRAPAGSGLPDLQISDAGLGVRNPAHIRPNGAAVSLPSGLATASTWDVDMARQA
GEMIGREAWLSGFNILLGGGADLTRDPRGGRNFEYAGEDPLQTGRMVGSTIAGIQSQHVISTLKHYAM
NDLETSRMTMSADIDPVAMRESDLLGFEIAIETGHPGSVMCSYNRVNDLYACENPYLLNTTLKQDWH
YPGFVMSDWGATHSSARAALAGLDQESAGDHADARPYFTALLAADVKAGRVPVARIDDMAQRIVRS
LFAAGLVAHPPQRGPLDVVTDTLVAQRDEEEGAVLLRNEGGILPLSPTARIAVIGGHADAGVISGGGSS
QVDPIGGEAVKGPGKKEWPGDPVYFPSSPLKAMRAEAPNAHITYESGTNIAAAVRAARAADVAVVYA
TQFTFEGMDAPSMHLDANADALITAVAAANPRTVVVMETGDPVLMPWNSSVAGVLEAWFPGSGGG
PAIARLLFGKVAPSGHLTMTFPQAESQLAHPDIAGVTADNVFEMQFKTDQELVYDEGSDVGYRWFDR
NHLKPLYPFGYGLTYTTFSTDGLAVHRHHDAVTVTFTVHNTGNRPGVDVPQVYVGLPDGGARRLAG
WQRVSLAPGESRAVTVQLDPRLLAHFDGKKDRWSIPSGTFRLWLGTSATDDSQQASLHLSGRTFAP

>bglX2 (encoding gene at position 522700:524739 bp)

MKYASLRYGLSALRCLIALCMGGLVLSALPVQARAAAGGSAGSAHAADMAARMQPAEQQAVLQAR
PIGGGEGATQAAADGLLAWPGVARLGLPALRMPDFSSPARPADEGGHPMLPWLALAATWDPDLARR
AGGARARAEWEQGRAVVRVGGIDLLPSGRLQAGEDQVLAGTMAGMIGAGLLSGHVLPVFGSVAGDE
QARADGAVPGRTAPVPAAWLADGSLLGVAMALEVAHGGALLCGGLAVCQTVTGLGAMVHDAWH
FPGMVMATPGGLGPARPDDASLLPGALGTGVDMEQVSSREGGHDMFGAPLRQAVRDGAVRPAQLQ
AMGMHVLASFYMAGSLDHPPAFTPAHAAASPPVADTLVSDVEAEGAVLLQNENRVLPFDPALGPVL
VLVRAPMRHAADLLADELRHAGLGVSVVVAADGANAPGTLPPATAQAARTLVLSGTDADNRMISTL
ADLGGHVVVVLTGDDPDRQMPWLDMVDSVVQAWAWRDAHARPLAALLSGQQEFSGHLPVTLTGG
FYPPGMVDADYRAFERAHVAPLFPFGYGLSARGHLTLSDLHVVREGTHLNASFSVTNPDGTAVRAIPQ
LYVSPSSDQAAPRRLAAWRSIMVAPGHTLRLTLPVSLRLLAQWNAPAANWAVAAGDYAFSLGFSSVM
LVRHADVRLPAMHLPAALDMAAD

>bcsAB2 (encoding gene at position 1020008:1024567 bp)

MAQFVDKITRFMEQAATGRVPSWVPIVIGVVMMSFVGSVALLPDMQGLISIGTVLLLLVLNRFKGRGITI
FLMMLSLLVSMRYVVWRLTSTIEFHGWIQSALSILLLLAEVYALSTLCLSYFQMAWPLGRKEHPLPEDTS
SWPHVDIYVPSYNEELSLVRSTVLGALKLDWPEDKLHVYILDDGRRVAFRDFALEAGAGYIIRSQNNHA
KAGNLNHALKITDGQFAVIFDCDHVPTRGFLKRTIGWMIADPKLALLQTPHHFYAPDPFQRNLVAGA
HVPPEGNMFYGLVQDGNDFWDATFFCGSCAVIRRSAVLGIGGFATETVTEDAHTALKMQRRGWRTA
YLREPLAGGLATERLILHIGQRVRWARGMLQIMRVDNPLLGRGLRWEQRLCYLSAMSHFLFAIPRVTFL
VSPLAYLFLGQNIIAASPLAISVYALPHIFHSILTLSRIESRWRYSFWSEIYETSLALFLVRITIVTLLQPHKGT
FNVTDKGGLLEKSYFDVGAVYPNVILAVILFAAFLRGIFGIVWQFHDRLALQSFALNTLWVVISLIIVLAS
IAVGRETRQTRAAPRIAVALPVRITDMEGRSFAGHTRDISLGGLGVDLHWPADVARPDRVMMEYVNE
RDGIHATVPATVLALDERSMRLQWERRDLEDESQIVDMVFGRNDAWANWADFEPDRPLRSIAMVLRS
IGGLFKWQAREIPRHVADDEEAKAPVVEAKLEKQSLVLKPVRRSARNGAAASAVLLVALAALSPVAM
AQVQPAASASVPDQTGVSAETPFGDSNTGTVPDMIPVIDQAAADRISDSEVTRTLSFRNLGATSGPLTLR
GYSPLQGLDVIVPANRVVTHARLSISGALSPSLLPEASAVTVTLNEQYIGTIKVDPEHPQFGPLTFDIDPLY
FTGDNKLNFRFAGEYRRDCNDLFNEILWARISDMSQITLTTVRIAPERKLSRLPAPFFDPNLRSTLRVPVV
LPDTNSKSSLKAAGLVASWFGKIADFRKLSFPVFTAIPASGNAVEVGENLPVDETGTRPRGPMLAEIAN
PNDRWGTILVVTGRNAQEVEAAARSLVFSADTLGGVAAKVVDDVSLEARQPYDAPAFVPTDRLVRFG
ELVGAADLQGGGFTPAGMTLPFHLPPDLYTWRGRPFLMDMWIRAPGGPVVDLETSRVDVSLNNNYL
QSYTLSPPGLWQRWSQRLVNLHAGAVGHTTALPTWLLFGQNQLLFNFDARPIDRGACRRTPGDIRMS
VDSDSTLDFRRGYHFAELPNLSYFAEAAFPFSRMADLSETTVVLPSHVDAGTAGAFLDLMGFFGASTW
YPAAGVQIMTADQVGAAQPGGDIVVLGTAEQLGSATSLLSRSPYVIRDGRIQVGEHMGLQGIWYLFQD
RDHAGLKNGVTARLNAPIVEAGLMLAAQSPYDHARSVVAFTGDTPERIHDLVLSLRNKTDLPSLQGDL
VIKNGNQFTSYRTAPTYTVGSLPLWVRADWYLSHHPLALYLTGLAGAALTALGVWGWLRRLSRRRIE
NEELAGEQ

>bcsC2 (encoding gene at position 1026603:1030553 bp)

MTRQYCPARGRQPAVAWRKAPAAWVMRPAACRPSSAWLAGGGWKIICGMVAGVIMAGEIAQAEPA
WTAGPDSAASPAGGSTQITVTPDAGQNAANAHLAHAAAVLELLLNQGYYWLGQHNLPKARETIQR
ALSIEPDNNEALFLLGRLQMAEGQTKLAAATLGRLIQNGNAPGLVADLRAQIHAGPIDPRGLAEARAL
AAEGKMMPAMFKYRALFKNGDPPPDLAMEYYRVLGATTLGYQEAATRLAAWVARNPRDLDAKLSL
DRILTYHVTSRDEGLEGLRQLARSNASAAIRDGAIAAWRDALLWEPVTGPTIALYNEWLDLHPGDAEII
DRRHKAQDAQNIIDGANYRQQGFVLLSRRNIEGAADLFHRALAINAHDADSLGGMALVAQARQQPA
LARHYFQQAIQADPDSAAHWRAALKAMEAGGGGGMDPLVARIIQAINSGHYDAAQSDLALLGRRG
NTITLQALQGMLARRQGHMEEAERLYREILRRAPGNADALFNLGGILIETGREAEAQDIIARLAHIRPD
LARHLEVAGLSARSDRTRNNDEKLSLLNRAMAMAPTDPWIRLKLARALDEAGNHAQAQAIMDGVTS
GRAVTPDDLQAAILYAMGRHDMARAEQLLARLPPGIESPGMARVAEQIELVRRIQELNRVPRAPNALL
VALADRPDPTGERGMRIANALLDRHAPQDAQQVLATEERLTQPPQPSQLLAYAGVYLRLHSAVDATR
CLNAFDAMARARPTDITADQQEIRNQIAIGLAIMTADGFNRYGQTARAYQVLAPVLQAHPDSAEAHL
AMGRVYQTRNMARRALEEDQIALRLKPHNIYALAAAARDAGGLHQMAEAKDYSTRLAHEDPDGPM
SWEVRSDIERIEGNTRAQLVDVEHARHAQCTLDGEGTCDGQHESFLPDYRWPEIDSNYINLHGATLPA
TYHYIPEDDGPEAMDRQIVYLRDSVSPQIDANSYVRSRTGTAGLGQLTEFAVPITGTLPFESWEHRLSFSI
VPTLLFTGNPLANPYSAHEFGTYAINGALPGSSHHYYTQGVGLSLNYVNHWFSADVGSSPLGFPITNVV
GGVEFAPRLTRNLGLRISGGRRMVTDSELSYAGMRDPGTGRLWGGVTRMFGHGALEWSEPTWNLYA

GGGFAYLGGTHVVDNTEVEAGAGGSATVWQSHDRQWLRVGLDLMYFGYKRDTYSFTWGQGGYFSP
QQYYGAMIPVEWSGHDRRWTWFLRGEAGFQHYHSNGAPYYPTDSALQALSVAHQPDYYGDEGESGL
AGNIRGRVVYQFTHRLRLGMEGGYSRAGNWSETSGMWMAHYTFDGQ

>bcsAB3 (encoding gene at position 2139998:2144443 bp)

MIWRILKSPFVTGPLFALLMAVVSLTYLTPDHQFFVAVGGGVLFFLVRRHDERWSRCFLMVLSIVVSGR
YLVWRFTSTLDLDGVLQGSLVLALAVGEIYTTIRVGFTYFQLAWPLRRQIHPLPEDEGTWPVIDVYVPTY
NEDISIVRTTVLGCLSMDWPADRLNVYILDDGRRRAFRDFAGQVGAGYINRAENVHAKAGNLNHAIG
VTSGDIIAIFDCDHVPVRGFLKKTVGWMLADPNLALLQTPHHFYSPDPFRRNMSRGMQVPPESNLFYG
LLQDGNDFWNATFFCGSCALLRRRAIVSINGFATETVTEDAHTALRMQRKGWGTGYLREPLAAGLETE
TLLLQIGQRVRWARGMFQMLRIDNPMLGRGLRLTQRICYMAAATNYFFAIPRMMFLLAPLAYLFLGVT
MIAASPYELAVYALPHLFHTTMTMSRLQGRWRYSFWSEIYESMLAPFLVRMTFVTMIAPHKGSFNVTD
KGGLLDRERFDWRASYPGVIVAVVLAVGLVSGIWAAIVHYHETLVFRAMAVNSIWVLFSLIIVLGGVA
AARETRQRRHSHRVAASLPFELVDAQGQVHACRSVDVSMGGCQLDAATVPGAAGGRVMLRWSLPS
GPATMGATIIARCGGRLHLQWVIAGLASERQVVALVFGRDDAWARWSDFPPDRPLRSLYLLVASICAL
FRPPPRVRDETPHAVARKTAAQDETLPRQQLVIPPDRTVVLRDIGTALVLAAMLLLPAMDARAQAAP
AMADAGPAVVADGSVDAIDNAHMTSADVDEVSHQPVTRTRTLADMGRTEDMVLRANAPLHGLSAG
VGRDIIITAARLGISGTARGAAGRVAVAVSINNQDAGVICPTADGAFGPVDLPLSPMFLDTRNRINFRLF
LRAPQPADPSAPACGPDARGRDGSAAQVSDPGVQVVISPRSTLTLTTVGLVPHRLLSALPYPVLDPDAA
RTAAVTFVLPGGRDPAQLEAAGMVASWLGLQSRESGIHFNVAAIPPDQGSAVAIAPGQPGPWGRAPA
GPALAVMPNPHDRFGTVLVVTGRTPAEVRTAAQALVLGAEHDAPGAFAAAPVVHPPARQPYDAPGV
VRTDRPVTLRELVSTAALRTRGLTSGTLDIALSLPPDLRSWRSRPFMARLQISAPEGGVLDRAKSRVSITL
NGTYLHSYPLVPLSLNPFRTPGAPVEHDLELPVWKMARHNELRLYFDVHPRHMTDAAAAASDAVVE
LDPSSTIDFSKSRHFAVLPDVALFAASAFPFSRMADLSETTVLLPPHPADGTVGAFVDMMGFAGAISRYP
ATGVTVRATDMPVDASVTGDILLLSTLDGLGNGRAALARAGCVRAPSLLARLRARVTQGRDVPACDF
TQGALVAAQSPFAAQRSVVAVLGGTPDALSAMVRDLRDPADTPRFQGDMVIRHGARLDSYRTGGLYT
VGNMPAWMLPDWYLGGHPLLLCGLGGLAALCGTSCAMRVLGARSRRRILNDDLTGDL

>bcsC3 (encoding gene at position 2136184:2139981 bp)

MPDGFAMPCRAFMPVGRPVRARMWGGVVLAGALCLYMPRAEAQTPLRYAETILDGQIEEGAFWIHH
GDDMHALHALQRALRIEPDNLEAGLMLGAVQVHQNDMAGARATLQALQAQPGAQAQVAALRGW
IGQAPIDPAALANARSAADTGKKLQATLLYRAVFQGAHPLPDMELEYDRVLSGSLSGYTEATQRLRAL
GALLPHDLEIRMALAQALSYRPATRPDAIEDMRQLATAATTPDFIRGEVMQSWRRTLEWMGADPQAE
PYYQEWLALHPDDTEIASRLKAEQAARAQAARLALVGAGYHALAHGELEQAERDFSSSMGTDPPRPE
ALEGLGLVAQRRGDIPAARRWLEQARALAPDDAGIRNALAGLDAPGGDPQLARLWALVARHQYDE
AQAMLPAVEKDHGRIADTLRVRAIIAHARHELPRAEAAWRAVLRLVPGDLPAAAALSDVLIEEGRITE
AEGWVARLRAAHYPGVTGLEAGVLGAMAENEPDQARRALLLEQALRDAPHNGWLRLHLAQLWLA
RGQATRARALMAPLCNLPPKTDEDTQVCFAFALQDQDMPRADALLARLPRADITAQMADGVAQVR
LWHQISHLPPDDAQAVPMLEHMPVVPDPEGTQARLVVNALLTRHAPVTAAAGVLHRALADSAGHV
SVNQSLSYAGLFMQIDDPVAAQQVLDGLPAVARGRRLTPTQARDLRDMTRSLAIAQADRDDIGGHPD
AARRLLDPLLAQGPDDADLLLARGRVEAALRQPAQAVVYDQKALAIRPDDTMAQAALARDSLASGH
EQAARAMARTLQATHPQWGDTWEIQAEIAGLDGRDRRRLADVQRARALDCTPRDDGDDSTGYGTRI

DAGCAPYRAHAGDEWPDIGTNFVPGMGAGMPEVYHYDPSLTPVQALDRQADYLQRALVPQADGNL
EIRDRSGQPGLGHMTVVNIPMTAIVPFSSTQHQLALSVMPSVLMSGDPLASGATAQQYGSVAAGGVRP
GFHVPAAVAGVALSAHYQWRWVAADVGSTPLGFTTTNVLGGIELAPHLTHSLTLRLTGERRAVTDSL
LAYSGARDPATGRVWGGVTRNRGHGQLEWAQPGYNLYAGGGYAVMQGTHTVANHEAEAGAGGS
GLLWHRQDAQHLRLGLDLVYFGYRRNTYFFTWGQGGYFSPHAFMAALVPLTYDGHAGRWTWLFRG
EAGYQHYTEHATAMFPLGEGGAMAGQRYAGQSTGGLAGNVLARAVYQLTPALRLGVEGGYSRSGS
WDEVHGMLMLHYAPG

>bcsAB4 (encoding gene at position 2248494:2253065 bp)

MLRELWLKWGGEALLTTPRRQAWLIALLMVVGAVLCVSVGDITLAPAEQAYISIGTITLFFILNRRPGR
HITCILMMLSLFVSFRYLIWRLGSTVEFHGPLQVAMSLALLGAEGYALSTLCLSYFQMSWPLGRKPHRLP
DDPADWPMVDVYVPSYNEDLELVRSTVLGAMDLHWPADKLNVYILDDGRRKAFRDFAVESGAGYIIR
AENNHAKAGNLNHAMQITKGEFVVIFDCDHVPTRSFLLKTIGWLWADPKLALLQTPHHFYSPDPFQR
NLAAGYDVPPEGNMFYGLIQDGNDFWDATFFCGSCAAIRRSALLSVGGFATETVTEDAHTALKMQRK
GWGTAYLCQPLAAGLATERLILHIGQRVRWARGMLQIMRRDNPMLGGGLRWEQRLCYLSAMSHFLF
AIPRVTFLVSPLGFLFFGQNIIAASPFAIMVYALPHIFHSIMTLSRIEGRWRYSFWSEIYETSLALFLIRITIVT
LLQPHKGKFNVTDKGGLLARGYFDFSAVYPNAIMAIILFGGMVRGIWGMIFQYHQKLAFQSFALNTL
WITVSLIVVLASIAVGRETRQIRHKPRVRVKLPVEVCFADGRVFQAHTTDISLGGAGVTMHLPEQIETPV
DITLRYSKPDDGIDIAVPARILGQQRGSWLHLQWKLDTLEEEREVVSLVFGRSDAWHNWADFKDDRPL
NSIYQVIKSIGGLLAPPYLWSLPSKGQEESSESVHKEETLEKKSLVIPPVPHRLGTGIIMLLVGAMLAAIPA
RAQMPPGYSHTGVSHLTPLGDTNSGDIPPATDTLDQKLADRVADAEITRTIAFRDLSRFPGPLTLRGFSP
LQGVDVVVPANRVVTHATLHLSGAISPSLLPEATAITVTLNEQYVGTIRIDPAHPTFGPIEFSIDPLYFTGD
SKLNFRFAGEYRRDCNDLYNDVLWAQISDQSTITLTTARIEPVRSLSRLPTPFFDSNLKVAMRVPMVLPG
SAMAPEVLRAGGLVASWFGRLADTRQLTFPVARTLPDTGNAIEIGPRIAIDDRGTPPSGPTLFEIANPND
KWGTILVVTGRTPAEVEVAARTLVFSPDTLGDLPGAVVEDVTLRPRVAYDAPSFIPTDRPVRFGELVTA
GDLQAGGFAPDTLHVPFHLPPDLYTWHRLPFLMDLWVRAPGNPVVDIAASRLDVSVNNTYIQSYSLL
NNNLWRIWSERMVTEHAGAVGHVTAIPPWLLFGQNDLQFQFDTRPVERGACRRTPDSPHLEIDSDSV
LDFRRGVHFTELPNLSYFAEIGFPFSRMADLGETTVVLPPAPDTDTIGAYFDLMGFFGSVTWYPVSGIHL
ATTEDIAHTPPEGDVILLAPVGQMGAAATLLSRSAYRIDGDHIRVGQQTGLQGIWYIFQDRDGSGLRN
GVTANLDMPVRNAALMIGSESPYASHRSVLALVGDNGIRIHELVASLHDPKVLPTIQGDLVIKNGEHFT
TYRTSPTYTVGHLPWWMWVDWFLSRHPMLMYLCGVTGAVMLGTGAWLWLRARARRRVKAQEKLD
EETARGEHH

>NAD-dependent malic enzyme (encoding gene at position 1377838:1379562 bp)

MSDTAHFPGQVPLHTALSGRELLDCPVLNKGNAFDRRERDLFGLHGLLPARIATLAEQVDLACMRLA
ALPDNFARHIALREIQDRNETLFYAIIDQAPESWLPIIYTPAIGRACQQFSQIWTRPRGLFLNYTDRGRIA
EILASPQWDGVRVIVASDGGSILGIGDQGANGMAIPIGKLSLYTACGGLDPACALPVLLDVGTDNAALL
ADPDYIGWRHARVRGAEYDAFIAEFVTAVAARWPDIALHWEDLSGADALRILRRYRGSLCTYNDDIQ
GTAGVTAGALLAAIRAGAAPLADQRIVIFGAGGAGCGIADLLAQMMVRAGMAAHEARSRFFMVDIN
GLVRHGMEGVTEGQMPFAQPADVAADWQVADPAHVTLAEVMAHVCPTTLIGTSGQGGAFTRQIVA
PMARQAARPVVFALSNPTANIEALPADLLEWTDGRAIIGTGGPFAPVTHAGRTRPVDQINNSYVFPGV

GLAVMAGGITRMTDGMFLAAADALAGLSPAVGAGGGHDAALLPPVSALRPVAMAVARAVIRQGQA
EGVAPAMPETGIGAMLAAAMWSPRYRPYEKTPPARP

>Pyruvate phosphate dikinase (encoding gene at position 2567253:2569928 bp)

MTKWVYSFGDGLNEGRADMRNLLGGKGANLAEMAANGLPVPPGFTITTEVCSAFYENGRKYPDELK
AQVDAALQRVEQSMGLRFGDPEAPLLVSVRSGARVSMPGMMDTVLNLGLNDQTVEGLARSSGDARF
AWDSYRRFIQMYGSVVMGVPHHHFEDVLEQFKRANNVEDDTAITAGQWRAIVVDYTHIITTHTGTDF
PTNPQDQLWGAIGAVFGSWMNPRANTYRKLHEIPASWGTAVNVQSMVFGNMGDDCATGVCFTRDP
STGENIFYGEYLINAQGEDVVAGIRTPQPMACARAEAGQHPMETTLPQAYAELLRVRSILETHYKDMQ
DIEFTVQRNVLYILQTRNGKRTAAAALKIAIDMAREGLITQEEAIQRVPAASLDQLLHPTLDPRAERVQL
TRGLPASPGAAAGAVVFTAEECEARAAKGEDVILVRIETSPEDVHGMHAARGVLTTRGGMTSHAAVV
ARGMGRVCVAGAGGIHVDYAAGSLSVGSHTVKEGEWITLDGGTGAVYLGRVPTIEPVLSDDFNTLMG
WADGVRRLGVRANAETPDDARTARRFGAEGIGLARTEHMFFGPDRIGFVRQMIIADEESVRQKAIAAL
LPFQREDFASLFRIMAGLPVTVRLLDPPLHEFLPHEAAEMAEVAQALGRSVEDVRARCAALAETNPML
GHRGCRLGLTSPEIYAMQVRALIQAAVLVEKELGRPIHPEIMIPLVATKAELATTRRAAEDEIARVLKEE
GTNLSYSIGTMIELPRAAIQADQIAEYADFFSFGTNDLTQTTFGLSRDDAGSFLPYYVDQGLLPRDPFVSI
DRDGVGALVRLGVERGRQTSADLKLGICGEHGGDPDSIAFFDEVGLDYVSCSPFRVPVARLAAAQAAL
ATRQKAASPA
